# Hypervirulent *Listeria monocytogenes* clones’ adaption to mammalian gut accounts for their association with dairy products

**DOI:** 10.1038/s41467-019-10380-0

**Published:** 2019-06-06

**Authors:** Mylène M. Maury, Hélène Bracq-Dieye, Lei Huang, Guillaume Vales, Morgane Lavina, Pierre Thouvenot, Olivier Disson, Alexandre Leclercq, Sylvain Brisse, Marc Lecuit

**Affiliations:** 1 Biology of Infection Unit, Inserm U1117, Institut Pasteur, 75015, Paris, France; 2 National Reference Centre and WHO Collaborating Centre for Listeria, Institut Pasteur, 75015, Paris, France; 3 Microbial Evolutionary Genomics Unit, CNRS UMR 3525, Institut Pasteur, 75015, Paris, France; 4 Université Paris Diderot, Université de Paris, 75013, Paris, France; 5 Paris Descartes University, Institut Imagine, Necker-Enfants Malades University Hospital, Division of Infectious Diseases and Tropical Medicine, APHP, 75006, Paris, France; 6 Present Address: Biodiversity and Epidemiology of Bacterial Pathogens Unit, Institut Pasteur, 75015, Paris, France

**Keywords:** Microbial ecology, Bacterial genetics, Pathogens

## Abstract

*Listeria monocytogenes* (*Lm*) is a major human and animal foodborne pathogen. Here we show that hypervirulent *Lm* clones, particularly CC1, are strongly associated with dairy products, whereas hypovirulent clones, CC9 and CC121, are associated with meat products. Clone adaptation to distinct ecological niches and/or different food products contamination routes may account for this uneven distribution. Indeed, hypervirulent clones colonize better the intestinal lumen and invade more intestinal tissues than hypovirulent ones, reflecting their adaption to host environment. Conversely, hypovirulent clones are adapted to food processing environments, with a higher prevalence of stress resistance and benzalkonium chloride tolerance genes and a higher survival and biofilm formation capacity in presence of sub-lethal benzalkonium chloride concentrations. *Lm* virulence heterogeneity therefore reflects the diversity of the ecological niches in which it evolves. These results also have important public health implications and may help in reducing food contamination and improving food consumption recommendations to at-risk populations.

## Abstract (web summary)

Here, Maury et al. show that hypervirulent *Listeria monocytogenes* (*Lm*) clones associated to dairy products exhibit higher adaptation to the mammalian gut environment, while hypovirulent clones persist in food processing environment, suggesting a relationship between *Lm* pathogenic potential and niche adaptation.

## Introduction

L*isteria monocytogenes* (*Lm*) is a ubiquitous foodborne pathogen that can cause a severe invasive infection in human and animals called listeriosis. In humans, it occurs mostly in immunocompromised individuals, the elderlies and pregnant women, and has one of the highest case fatality rate among foodborne pathogens (20–30%)^1^. Human listeriosis manifests mostly as septicemia, central nervous system infections, and maternal–neonatal infections leading to major fetal or neonatal complications in 80% of the cases^1^. In animals, especially in ruminants, it induces rhombencephalitis and abortions, and less frequently mastitis, which is often undiagnosed owing to the frequent absence of associated symptoms^2^. Although very little is known about *Lm* fecal carriage in human, it occurs in clinically healthy ruminants, and correlates with *Lm* fecal shedding in the environment^3–5^. Indeed, a high prevalence of *Lm* has been observed in fecal samples of ruminant herds (e.g., 46.3% of dairy cattle, 30.6% of beef cattle, and 14.2% of sheep herds were contaminated in a total of 343 studied herds^5^).

*Lm* is a genetically heterogeneous species in which isolates can be grouped into lineages^6,7^, PCR serogroups^8^, multilocus sequence typing (MLST) clonal complexes (CCs or clones)^9^ and core genome MLST (cgMLST) sublineages and types (CTs)^10^. A considerable heterogeneity in pathogenic potential among *Lm* isolates has been observed^11–15^. In particular, hypervirulent MLST clones with high clinical frequency have been identified, such as CC1, CC2, CC4, and CC6^15^. CC1 is also highly associated with *Lm*-associated rhombencephalitis in ruminants^16^, and the analysis of fecal samples of beef cattle and sheep herds has shown that serotype 4b (which includes CC1) is the most prevalent^5^. Together, these data highlight the high virulence of this clone for cattle and its presence in farm environments. In contrast, CC9 and CC121 are associated to a food origin, are hypovirulent, and infect mostly highly immunocompromised individuals^15^, in part owing to truncations in InlA, a major *Lm* virulence factor^17^ involved in the crossing of host barriers^18,19^. In addition, CC121 has been shown to persist in food production environment^20,21^.

Because of the severity of human listeriosis, many countries have established surveillance systems to rapidly identify and recall contaminated food products^22–24^. Analysis of long-term surveillance data has shown that both raw and processed food products can be contaminated by *Lm* at different production stages^25–27^, and that some food categories are more frequently contaminated^28^, including dairy products^29^, meat products^30^, seafood^31^ and mixed ready-to-eat products^32^.

The large panel of food products that can be contaminated by *Lm* and its ubiquitous distribution in the environment reflect the unique capacity of this bacterial species to survive, multiply, and/or produce biofilm in a wide range of conditions, including low temperatures^33^, acidic conditions^34^, high salinity^35^, or in presence of commonly used surface disinfectants, such as benzalkonium chloride (BC)^20,21,36^. In order to cope with these extreme conditions, stress resistance determinants have been selected in *Lm*, conferring resistance to environmental stresses, such as low pH, high osmolarity, bile and nisin (SSI1)^37,38^, cadmium and arsenic (LGI-2)^39^, and alkaline and oxidative stresses (SSI2)^40^. In addition, several BC tolerance determinants have been identified in *Lm*, including BC efflux pumps *qac* (Tn6188)^10,41,42^, *bcrABC*^43^, and *emrE*^44^, which are mostly present in lineage II isolates and more specifically in CC121, CC9, CC31, CC13, and CC14^10,20,21,36^. Additional BC tolerance genes have been identified, such as *emrC*, identified on a plasmid in some ST6 isolates^45^, *qacA*^46^, and *qacC*^47,48^, which are both located on plasmids, and *mdrL* (*lmo1409*), negatively regulated by *ladR* (*lmo1408*)^49^.

Given the striking differences of pathogenic potential among *Lm* clones^15^, it is of major interest to investigate their distribution in different types of food products, and in particular, test for association of hypervirulent and hypovirulent clones with distinct food categories. Indeed, this may help to better characterize the ecological niches of this pathogenic bacterium and inform on the external conditions in which *Lm* evolves both its virulence potential and environmental survival capacities. These data may also help understanding how *Lm* circulates between animals, food/feed, humans, and the environment and eventually help better identify food contamination routes, leading to a reduction of food contaminations and to a better prevention of human listeriosis. The objectives of this study are (i) to identify association of *Lm* CCs to particular food sources, and specifically to test whether hypervirulent and hypovirulent clones are associated to certain food types; and (ii) to identify bacterial factors that may explain the contrasted distribution of clones in food sources. Here, we show that hypervirulent clones are associated with dairy products and colonize better the intestinal lumen and invade more intestinal tissues than hypovirulent ones. In contrast, hypovirulent clones are associated with meat products and exhibit a higher prevalence of stress resistance and BC tolerance genes, and a higher survival and biofilm formation capacity in presence of sub-lethal BC concentrations.

## Results

### Uneven distribution of *Lm* clones in food products

**Fig. 1:**
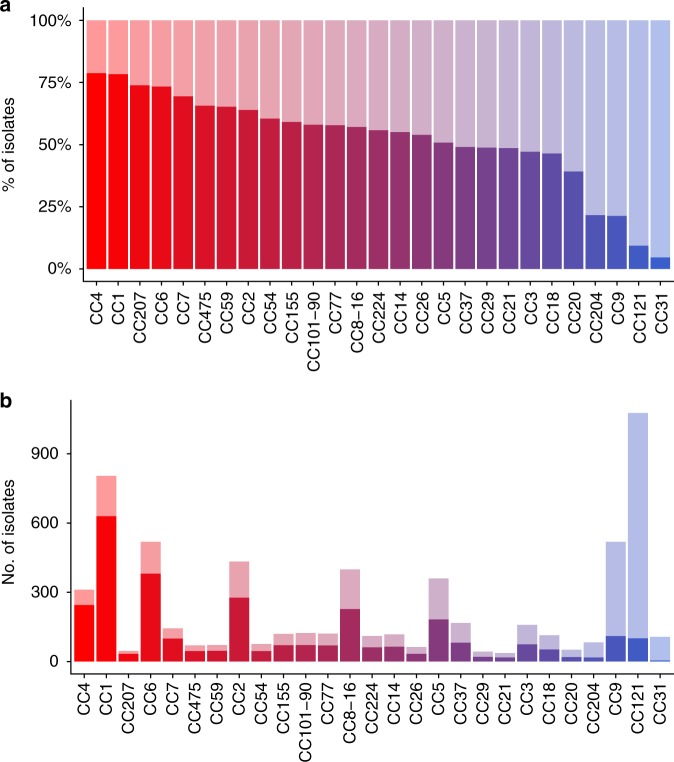
Distribution of *Lm* CCs in food and clinical sources. A total of 6641 non-redundant *Lm* isolates collected between January 2005 and May 2016 by the National Reference Center for *Listeria* in the context of the epidemiological surveillance of listeriosis in France are represented in the figure. Percentages and numbers of food and clinical isolates per MLST clone are shown. Major CCs that represent individually at least 0.5% of all the isolates of the study are shown, representing in total 94.1% of all isolates. **a**, **b** Clinical isolates are represented in dark colors and food isolates in light colors per clone. **a** Clones are sorted by percentage of clinical isolates. The color gradient is set according to the clinical frequencies of the clones (red, high clinical frequencies; blue, low clinical frequencies). Colors defined in this graph are used in all the other figures of the article, where appropriate. **b** Number of isolates per clone from clinical and food sources are shown with the same ordering than in **a**

We studied the totality of the food (*n* = 3333) and clinical (*n* = 3308) non-redundant isolates prospectively collected for 12 consecutive years (from 2005 and 2016) in the context of the surveillance of listeriosis in France. Clinical isolates were collected with a high degree of exhaustiveness owing to mandatory declaration of listeriosis in France, and are therefore highly representative of the listeriosis cases that occurred in France during this time period^50^. Food isolates were collected concomitantly in the context of food alerts triggered when contaminated food products are on the market, own-checks performed by food industries, or in case of investigations following neurolisteriosis cases. Therefore, they are genuinely representative of the *Lm* isolates to which the whole population is exposed. As previously shown for a narrower time window (2005–2013), important differences of clinical frequency are observed among clones (Fig. 1a; Supplementary Data 1)^15^. Indeed, as we have previously reported, CC1, CC6, CC2, and CC4 have the highest clinical frequencies among numerically major clones, whereas CC9 and CC121 have the lowest clinical frequencies (Fig. 1a, b; Supplementary Data 1)^15^. Note that the colors for each CC in Fig. 1a are used throughout the manuscript in all figures, where appropriate.

Most food isolates (*n* = 1408) were from meat products (42.2%), 758 were from dairy products (22.7%), 406 from seafood products (12.2%), 354 from mixed products (10.6%), 103 from fruits and vegetables (3.1%), and 304 from unknown food sources (9.1%) (Supplementary Data 1).

**Fig. 2:**
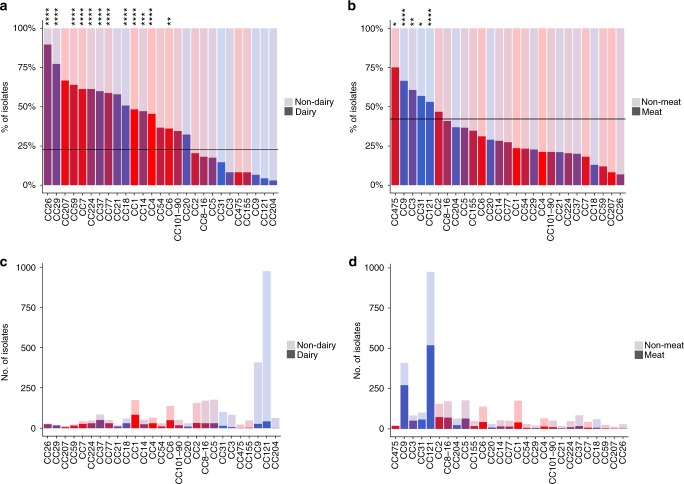
Distribution of *Lm* CCs in food categories. Percentages and numbers of isolates from dairy products and meat products per MLST clone are shown. Major CCs that represent individually at least 0.5% of all the isolates of the study are shown, representing in total 94.1% of all isolates. **a** Proportions of dairy (dark colors)/non-dairy (light colors) isolates per CC. Non-dairy isolates were those isolated from meat products, seafood products, fruits and vegetables, mixed products, and unknown products. Clones are sorted per percentage of isolates from dairy products. **b** Proportions of meat (dark colors)/non-meat (light colors) isolates per CC. Non-meat isolates were those isolated from dairy products, seafood products, fruits and vegetables, mixed products, and unknown products. Clones are sorted per percentage of isolates from meat products. **a**, **b** Horizontal lines represent the mean percentage of dairy products in **a** and meat products in **b** considering all food isolates together. Colors are as in Fig. 1a. Positive statistical associations (*χ*^*2*^ tests) of CCs with dairy or meat origins are shown. ****p < 1.10^−5^; ***p < 1.10^−4^; **p < 1.10^−3^; *p < 0.05. Statistics for mixed products, fruits and vegetables and unknown sources are not provided. **c** Number of dairy (dark colors) / non-dairy (light colors) isolates per clone in the same ordering than in **a**. **d** Number of meat (dark colors)/non-meat (light colors) isolates per clone in the same ordering than in **b**

**Fig. 3:**
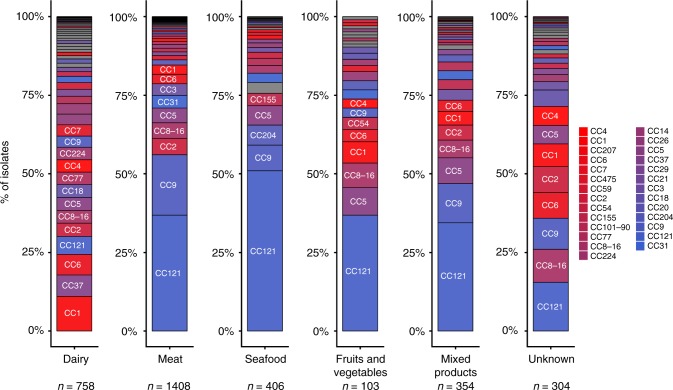
CC proportions in different food categories. The 3333 food isolates included in the study are represented. Percentage of isolates of each CC per food type (dairy products, meat products, seafood, fruits and vegetables, mixed products, and unknown food types) are represented. Major CCs representing individually at least 0.5% of all the isolates of the study are shown with similar colors than in Fig. 1a. The other CCs, considered as minor, are represented in gray. Clones relevant for interpretation are indicated on the graphs. Number of isolates from each food type are indicated below the graphs

Stricking differences were observed among clones when considering proportions of isolates from different food types (Fig. 2a, b; Supplementary Data 1). Indeed, only 4.4% of the CC121 food isolates were from dairy products (Fig. 2a, c; Supplementary Data 1), whereas 53.2% were from meat products and 21.2% from seafood products, with a strong association of this clone with meat and seafood products (*χ*^2^ test, *p* < 1.10^−5^; Fig. 2b, d; Supplementary Data 1). In addition, CC121 was the most prevalent CC in meat products (36.9%), seafood (51%), fruits and vegetables (36.9%), and mixed products (34.5%), whereas it represented only 5.7% of isolates from dairy products (Fig. 3; Supplementary Data 1). The other major hypovirulent clone CC9 was also rarely isolated from dairy products (6.6%) (Fig. 2a, c; Supplementary Data 1), but frequently isolated form meat products (66.4%) (Fig. 2b, d; Supplementary Data 1), with a strong association with this latter type of food (*χ*^2^ test, *p* < 1.10^−5^; Fig. 2b; Supplementary Data 1). CC9 was the second most represented clone in meat, seafood, and mixed products, corresponding to 19.2%, 8.1%, and 12.4% of these food sources, respectively, whereas it represented only 3.6% of isolates from dairy products (Fig. 3; Supplementary Data 1). Altogether, CC121 and CC9 represented around half of all isolates from meat, seafood and mixed products (Fig. 3). Of note, CC9 was rare in fruits/vegetables in contrast to CC121, as they represented 2.9% and 36.9% of isolates from this food type, respectively (Fig. 3; Supplementary Data 1). In sharp contrast, 48.3% of CC1 isolates were from dairy products, with a strong association of this clone with this food type (*χ*^2^ test, *p* < 1.10^−5^; Fig. 2a, c; Supplementary Data 1), whereas only 23.6% of CC1 isolates were from meat products (Fig. 2b, d; Supplementary Data 1). CC1 was the most frequent clone isolated from dairy products, representing 11.1% of all isolates, whereas it represented only 2.9% of isolates from meat products (Fig. 3; Supplementary Data 1). CC37 and CC6 were the second and third most frequent clones in dairy products, representing 6.7% and 6.6% of all dairy isolates, respectively.

These results highlight key differences in *Lm* clonal distribution in dairy products as compared with meat and other products. Altogether, these data show that the major hypervirulent clones CC1, CC4, and CC6, which are highly associated to human and animal clinical origins^15,16^, are associated with dairy products (*χ*^2^ test, *p* < 1.10^−5^ for CC1 and CC4; *p* < 1.10^−3^ for CC6) (Fig. 2a; Supplementary Data 1); whereas the major hypovirulent clones CC9 and CC121 are associated with meat products (Fig. 2b; Supplementary Data 1). In order to quantify the strength of the association between clonal distribution in dairy products, meat products and clinical origin, we performed weighted linear regressions (Supplementary Fig. 1a, b). This analysis demonstrates that the frequencies of clones in dairy products and clinical samples are linearly and positively correlated (weighted linear regression, *p* < 1.10^−7^). In contrast, clone prevalence in meat products is negatively correlated with their frequency in clinical samples (weighted linear regression, *p* < 1.10^−7^).

Overall, these differences suggest key differences of contamination modalities of dairy versus meat products and all other food categories, and/or differences in niche adaptation among CCs. Meat products are initially physiologically sterile, and are therefore most likely contaminated by *Lm* during processing and/or storage. In contrast, dairy products, which physiologically contain bacteria^51^, may be contaminated by *Lm* before and/or during milking. This hypothesis is supported by the observation that CC121 and CC9 rank among the least frequent clones in dairy products made of raw milk (ranks 17 and 20, respectively; Supplementary Fig. 2), whereas in dairy products made of pasteurized/unknown type of milk, these two clones are the second and the 7th most abundant clones, respectively (Supplementary Fig. 2), with an association of these clones with dairy products made of non-raw milk, as compared with the rest of the species (*χ*^2^ test; *p* = 0.042 for CC9; *p* = 0.002 for CC121). In addition, CC9 and CC121 are associated with products that need to be processed before consumption (e.g., meat, seafood; cf. above) and are known to often harbor BC tolerance genes that could be involved in their survival/growth and persistence in food production environments^10,20,21,36^.

### *Lm* persistence in food production environment

**Fig. 4:**
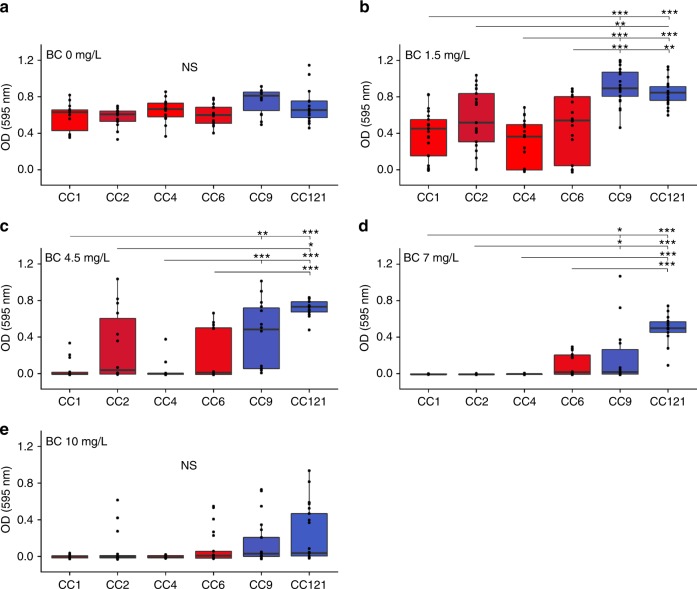
Biofilm formation efficiency in absence and presence of BC. Biofilm formation capacity in absence and presence of 1.5, 4.5, 7, and 10 mg/L of benzalkonium chloride (BC) is shown for 42 isolates representative of CC1, CC2, CC4, CC6, CC9, and CC121 (seven isolates per CC). Biofilm was quantified after 48 h of static growth in modified MCDB 202 medium with or without BC using a violet crystal labeling-based method and is shown as OD_595nm_ (see Methods section). Colors are as in Fig. 1a. The boxes delimit the first (25%) and third (75%) quartiles, and the bars delimit the second quartile (median). Outliers are outside the whiskers. Statistical differences between hypervirulent (CC1, CC2, CC4, and CC6) and hypovirulent (CC9 and CC121) clones are shown (Mann–Whitney *U* test with holm’s correction). ***p < 10^−3^; **p < 10^−2^; *p < 0.05. *n* = 7 strains per clone tested three times independently. NS: Not significant. BC: Benzalkonium chloride. Source data are provided as a Source Data file

We tested whether CC9 and CC121 would exhibit characteristics associated with a higher persistence in food processing environments as compared with CC1, CC2, CC4, and CC6, using a set of 42 isolates representative of each of these clones, based on their cgMLST profiles (Supplementary Data 2). To this end, we analyzed the relative capacity of *Lm* clones to grow and form biofilm^52,53^ at 37 °C in absence and presence of sub-lethal BC concentrations^54^. In absence of BC, although CC9 seemed to produce more biofilm than other clones, it was not statistically significant (Fig. 4a). Yet, upon increasing BC concentrations (1.5–7 mg/L), CC9 and CC121 produced significantly more biofilm than CC1, CC2, CC4, and CC6 (Fig. 4b–d). Median OD_595nm_ values were close to zero for CC1, CC2, CC4, and CC6 with 4.5 mg/L BC and above, whereas for CC9 and CC121 this point was reached at 7 and 10 mg/L BC, respectively (Supplementary Fig. 3 and Fig. 4d, e), showing that biofilm formation by CC9 and CC121 can occur in presence of higher BC concentrations than for CC1, CC2, CC4, and CC6. Regarding bacterial growth, an increased lag phase upon increasing BC concentrations was observed for some of the isolates, mostly from hypervirulent clones (Supplementary Fig. 4). Neither growth nor biofilm formation was observed for any analyzed isolate in presence of 200 mg/L of BC, which is lower than the concentrations commonly used in food production facilities^55^, suggesting that BC tolerance in *Lm* has an impact on its persistence only at sub-lethal BC concentrations, as already suggested^56^.

Because refrigeration conditions are used for food storage and are known to allow *Lm* growth, we tested whether *Lm* clones have distinct capacities to grow and form biofilm at 4 °C in presence and absence of BC. Although low biofilm formation was observed when bacteria were cultured at 4 °C for 20 days (median OD_595nm_ = 0.006), significantly more biofilm was formed by CC9, CC121, and CC4 in presence of 10 mg/L BC as compared with similar conditions without BC (Mann–Whitney *U* test, *p* < 0.05) (Supplementary Fig. 5a–f), suggesting that BC may favor biofilm formation by these isolates at 4 °C. In addition, CC9 and CC121 produced significantly more biofilm than CC1 and CC2 in presence of 10 mg/L BC (Supplementary Fig. 5g, h).

### Distribution of stress resistance genes in *Lm* clones

Among the tested isolates, none of those with an increased lag phase and reduced biofilm formation upon increasing BC concentrations harbored *qac* (Tn6188)^38,39^, *bcrABC*^42,57,58^*, emrE*^44^, *emrC*^45^, *qacC*^47,48^, or *qacA*^46^ (Supplementary Data 2). *mdrL* and *ladR* are present in the genome of every tested isolate, as they belong to *Lm* core genome^10,15^. None of the tested CC1 and CC4 isolates harbored BC tolerance genes, whereas all CC121 tested harbored *qac* (Tn6188) (Supplementary Data 2), which is consistent with previous reports^10,41,42^. *qac* was also present in three CC2 and two CC9 isolates, whereas two other CC9 isolates harbored *bcrABC*^43^ (Supplementary Data 2). Three CC6 isolates harbored the *emrC* gene^45^. No isolate harbored *emrE*^44^, *qacC*^47,48^, or *qacA*^46^. In order to assess the role of *qac* and *bcrABC* in the BC tolerance phenotypes observed in CC9 and CC121, we constructed a CLIP 2016/00360∆*qac* mutant (i.e., a CC121 strain where *qac* has been removed) and a complemented strain CLIP 2009/00521+pPL2::*bcrABC* (i.e., CC9 strain where a pPL2 plasmid harboring the *bcrABC* gene cassette under its native promoter has been inserted intra-chromosomally) and measured their OD_600nm_ after 2 h of growth in BHI with various BC concentrations. Both strains harboring no BC tolerance gene (CLIP 2009/00521WT and CLIP 2016/00360∆*qac*) exhibited a lower growth than strains harboring *qac* or *bcrABC* (CLIP 2016/00360WT and CLIP 2009/00521+pPL2::*bcrABC*, respectively) in presence of 4 mg/L of BC (Supplementary Fig. 6), confirming the role of *qac* and *bcrABC* in the observed BC tolerance^42,57,58^. BC concentration higher than 6 mg/L induced no difference of growth between isolates, showing that *qac* and *bcrABC* are only advantageous in presence of sub-lethal BC concentrations.

In order to gain a global view on the distribution of these genes among *Lm* clones, we analyzed all available genome sequences (*n* = 2928) of non-redundant *Lm* isolates collected from 2015 to 2018 in the context of the French surveillance of listeriosis. This revealed that 32.1% of all isolates harbor at least one of the known and non-core BC tolerance genes, namely *qac*, *bcrABC, emrC*, *qacC, emrE,* or *qacA*, which were present in 18.8%, 8.2%, 5%, 0.1%, 0.03%, and 0.03% of isolates, respectively (Supplementary Fig. 7). Interestingly, only 45.1% of the clones analyzed contained isolates harboring BC tolerance genes (32/71). Of the 98.1% of CC121 isolates harboring a BC tolerance gene, all harbored *qac*, and all other BC tolerance genes were absent from this clone. In CC9, 58.2% of isolates harbored at least one BC tolerance gene, which were more diverse than in CC121 (*qac*, 22.5%; *bcrABC*, 20.1%; *emrC*, 16.1%; both *qac,* and *bcrABC*, 0.4%). Only one CC9 isolate harbored both *qac* and *bcrABC*. Very few isolates of CC1 and CC4 contained BC tolerance genes (3.6% and 2.1%, respectively), whereas CC2 and CC6 showed a higher prevalence of these genes (43.6% and 14.1%, respectively). This is in line with our finding that within CC2 and CC6, a higher proportion of isolates are from meat products and a lower proportion from dairy products, as compared with CC1 and CC4 (Fig. 2a, b).

Together, these results show that BC tolerance is higher in hypovirulent CCs, owing to higher frequency of BC tolerance genes in these clones, leading to better growth and biofilm formation in presence of BC. This may confer a higher persistence of these clones in food production environments where BC is classically used and may remain at low concentrations after sanitation^56^.

**Fig. 5:**
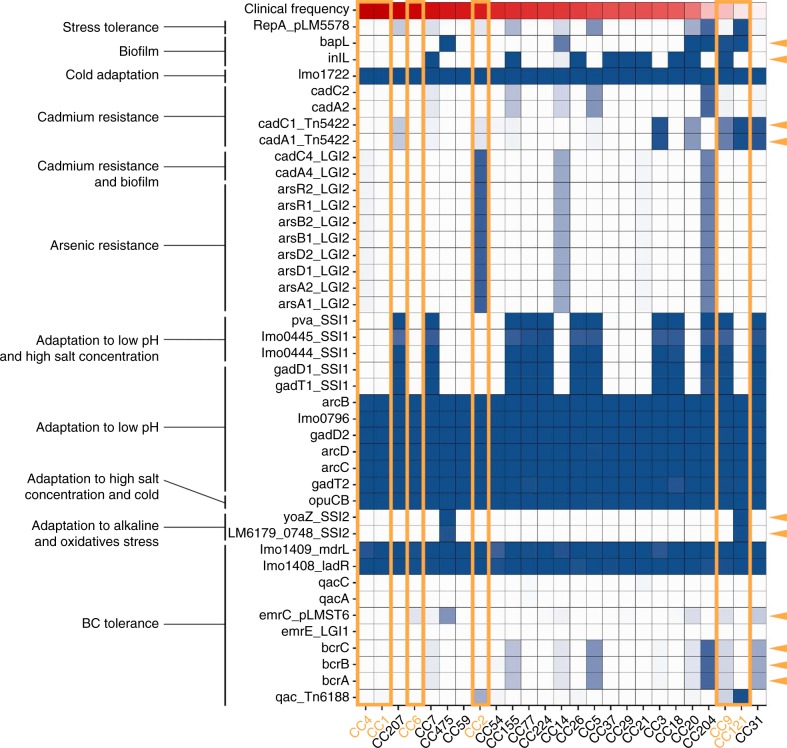
Prevalence of stress resistance genes in *Lm* clones. We analyzed a set of 2928 genome sequences of non-redundant *Lm* isolates collected from 2015 to 2018 in the context of the French surveillance of listeriosis. Major CCs representing individually at least 0.5% of all the isolates of the study are shown. Prevalence of genes known to be involved in *Lm* stress resistance is shown per clone^21^. The blue intensity reflects the percentages of isolates harboring the considered genes. Clones are sorted according to their clinical frequency, represented with a red gradient at the top of the graph. Clones analyzed with in vivo and in vitro experiments in this study (i.e., CC1, CC2, CC4, CC6, CC9, and CC121) are highlighted with orange rectangles. Genes are sorted according to their function. Orange arrows indicate genes that are enriched in clones having a low clinical frequency. LGI: *Listeria* genomic island. SSI: Stress survival islet. BC: Benzalkonium choride

In order to investigate the extent of stress adaptation of clones more globally, we analyzed all the non-core *Lm* genes known to be involved in tolerance to a large variety of stresses^21^. Interestingly, some genes involved in stress tolerance appear to be enriched in clones having a low clinical frequency, whereas clones that are more host associated show less stress resistance genes (Fig. 5). More specifically, while the hypervirulent CC1, CC4, and CC6 clones only harbor genes that are common to all clones; CC2 harboring also the *Listeria* genomic island 2 containing genes involved in cadmium and arsenic resistance; CC9 and CC121 harbor additional stress resistance genes that may help them to adapt to highly diverse stress conditions, including genes involved in biofilm formation (*bapL* and *inlL* for CC9, and *bapL* for CC121), cadmium resistance (*cadA1* and *cadC1*), adaptation to low pH and high salt concentration (SSI1 genes for CC9), adaptation to alkaline and oxidative stress (SSI2 genes for CC121) and BC tolerance (as shown above). In addition, clones having a lower clinical frequency seemed to harbor more frequently plasmids (i.e., CC121, CC204, and CC20), as assessed by the presence of a *repA* gene in these isolates. As plasmids are known to frequently contain stress resistance genes^45,58,59^, this may be advantageous for environmental adaptation of these clones. These results suggest that CC9 and CC121 are better adapted to environmental stress conditions than host-associated clones.

### Growth in different environmental conditions

Both within food products and after ingestion by a host, bacteria need to cope with many different stresses, including low pH and high osmolarity^60,61^. We therefore tested if clones differ in their capacities to survive and grow in acidic (pH 5–7) and salty (NaCl 0.09 m, basal NaCl content in BHI; and NaCl 1.1 m) conditions in BHI at 22 and 37 °C. At 37 °C and pH 6 and 7, adjunction of NaCl to BHI (1.1 m of total NaCl) induced a better growth of CC1, CC2, CC4, and CC6 than CC9 and CC121, as indicated by significantly higher areas under the growth curves (AUC) for the former as compared with the latter clones; whereas no difference was observed with NaCl 0.09 m (Supplementary Fig. 8 and 9).

At 22 °C, pH 6, and in presence of 1.1 m of NaCl, CC9 and CC121 grew significantly better than CC1, CC2, CC4, and CC6, as deduced from their AUC values (Supplementary Fig. 8 and 9). These differences suggest that CC9 and CC121 have a growth advantage in environments compatible with conditions used for food preservation, cheese ripening or meat preparation in food production plants.

### Adaptation to host environment

**Fig. 6:**
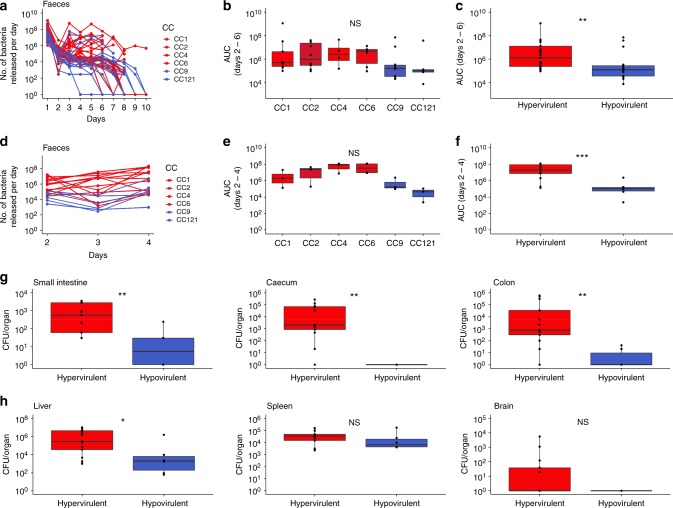
Gut colonization capacities of *Lm* clones. Colonization capacity of clones was assessed by orally inoculating mEcad E16P KI female C57BL/6J mice^62^ by mixes of seven isolates of the same CC (tested CCs: CC1, CC2, CC4, CC6, CC9, and CC121), and by enumerating the total bacteria released in mice stools every day (see Methods section). **a**–**c** Each mouse was orally infected by 2 × 10^7^ bacteria. *n* = a minimum of 4 mice per CC. **a** Number of bacteria of each CC released in mice stools per day. **b**, **c** Area under the curves of panel **a** per CC is shown in **b** and for hypervirulent (CC1, CC2, CC4 and CC6) and hypovirulent (CC9 and CC121) CCs in **c**, calculated for the interval between days 2 and 6. **d**–**h** Each mouse was orally infected by 2 × 10^8^ bacteria. *n* = a minimum of three mice per CC. **d** Number of bacteria of each CC released in mice stools per day. **e**, **f** Area under the curves of panel **d** per CC is shown in **e** and for hypervirulent (CC1, CC2, CC4, and CC6) and hypovirulent (CC9 and CC121) CCs in **f**, calculated for the interval between days 2 and 4. **g** CFU counts for intestinal organs (small intestine, cecum, and colon) treated with gentamicin (100 μg/mL; 2 h) of mice killed on the 4th day after inoculation. **h** CFU counts for deeper organs (liver, spleen, mesenteric lymph nodes, and brain) of mice killed on the 4th day after inoculation. Colors are as in Fig. 1a. In boxplots, the boxes delimit the first (25%) and third (75%) quartiles, whereas the bars delimit the second quartile (median). Outliers are outside the whiskers. Mann–Whitney *U* test was used for statistical comparisons. ***p < 1.10^−3^; **p < 1.10^−2^; *p < 0.05. NS: Not significant. AUC: Area under the curve. Source data are provided as a Source Data file

In contrast to CC9 and CC121, CC1 is hypervirulent in human^15^, and is also highly associated with infection of ruminants^16^ in which subclinical *Lm* infections and prolonged fecal carriage occur^2,5^. The over-representation of CC1 in dairy products may therefore be a consequence of a frequent undetected carriage of CC1 in ruminants, leading to more frequent presence of this clone in dairy cattle farms and dairy products as compared with the other clones. To test this hypothesis, colonization experiments were conducted in mice permissive to *Lm* oral infection^62^. mEcad E16P KI female mice were inoculated orally with a mix of seven genetically diverse isolates per clone (CC1, CC2, CC4, CC6, CC9, or CC121; 2 × 10^7^ bacteria/mouse in total, Supplementary Data 2), and stools were collected daily and subjected to bacterial enumeration. Fecal bacterial shedding (as assessed by AUC) between days 2 and 6 was slightly higher for CC1, CC2, CC4, and CC6 than for CC9 and CC121 but not significantly (Fig. 6a, b), whereas when considered together, fecalshedding of hypervirulent clones (CC1, CC2, CC4, and CC6) was significantly higher than hypovirulent clones (CC9 and CC121) (Mann–Whitney *U* test, *p* = 0.003; Fig. 6c). Overall, these results indicate that hypervirulent CCs are better gut colonizers than hypovirulent CCs. We showed in the previous paragraph that hypervirulent CCs grow better than hypovirulent ones in BHI at 37 °C, pH 7, in presence of NaCl 1.1 m. As salt is known to be present in high concentrations in the gut^60^, these results suggest that hypervirulent CCs may exhibit a better fitness than hypovirulent CCs in this environment, which may contribute to their greater capacity to colonize the gut (Fig. 6a–c).

In order to assess the level of infection of deeper organs, we infected mice orally with 2 × 10^8^ bacteria/mouse, collected stools daily and killed mice on day 4 after infection. Fecal shedding of *Lm* was consistent with the results obtained with a lower inoculum (Fig. 6d–f). Colony-forming units (CFU) counts of *Lm* bacteria within the small intestine, cecum, and colon tissues show that hypervirulent clones invade more efficiently the gut tissues than hypovirulent clones (Fig. 6g). These data suggest that hypervirulent clones CC1, CC2, CC4, and CC6 cross more efficiently the intestinal barrier, and part of these bacteria could be shed back in the gut lumen^63^, leading to a higher gut colonization, fecal shedding, release of *Lm* in the environment and ultimately favoring inter-host transmission of host-adapted strains. Deeper organs (liver, spleen, and brain) were also more infected by hypervirulent CCs than hypovirulent CCs, although the difference was only significant in the liver under the experimental conditions tested (4 days post infection, 2 × 10^8^ bacteria/mouse) (Fig. 6h).

### Gene association with dairy and meat origins

We next performed a genome-wide association study (GWAS) to identify genes associated with dairy or meat origins, in order to identify genes that may be involved in either host colonization or adaptation to food production environment, respectively. To this end, we first built the pangenome of 1129 genomes from isolates of dairy and meat origins (out of the 2928 total genomes analyzed above), and obtained a total of 21,546 gene families. We then looked for genes enriched in genomes from dairy or meat isolates (see Methods section). We obtained 455 and 916 gene families associated with a dairy and meat origin, respectively (Supplementary Data 3 and 4). Classification of these genes into Clusters of Orthologous Groups (COG) showed a distribution significantly different between the dairy- and the meat-associated genes (Fisher’s exact, *p* < 1.10^−3^) (Supplementary Fig. 10). Among the dairy-associated genes, those involved in cell wall/membrane/envelope biogenesis (M); carbohydrate transport and metabolism (G) and transcription (K) predominated, whereas meat-associated genes were mostly involved in transcription (K) and replication, recombination and repair (L) (Supplementary Fig. 10).

These differences may indicate the need for *Lm* to acquire new accessory genes to adapt to one or the other environment.

## Discussion

Understanding the distribution of *Lm* CCs in different food types has remained a key missing piece to fully unravel the biology of *Lm* with regard to its ecological niches adaptation. We show here that hypervirulent clones are strongly associated with dairy products and adapted to the mammalian gut environment, whereas, in sharp contrast, hypovirulent clones are associated to processed food products and persist in food-processing environment in a saprophytic lifestyle. This argues for a strong relationship between *Lm* pathogenic potential and ecological niche adaptation.

CC1 is hypervirulent in human^15^ and strongly associated with ruminant rhombencephalitis^16^. Here, we show that CC1 is highly over-represented in milk-derived products and that hypervirulent CCs including CC1 are better colonizers of the gut lumen and tissue than hypovirulent clones. This suggests that CC1 is more adapted for within-host survival, persistence, fecal shedding, and likely inter-host transmission, as compared with other clones. This underlines a correlation between virulence and fecal shedding, as previously proposed^63^, and may account for hypervirulence emergence and maintenance. In the particular case of dairy cattle, the higher gut colonization capacity of CC1 may lead to prolonged fecal shedding, resulting in its high prevalence and persistence in dairy cattle farm environments^4,64^. In healthy animals, *Lm* fecal shedding has been shown to occur to a high frequency^5^, and longitudinal studies have shown that a given *Lm* isolate can be excreted for months from animals in the absence of clinical symptoms^2^. Milk and milk-derived products may therefore be contaminated to high frequency owing to *Lm* carriage in ruminants (10–16% of contaminated bulk tank milk samples in a total of 186 tested samples^65^). Re-ingestion of feed contaminated by fecal materials (fecal–oral cycle), more frequently by CC1, may contribute to maintain and amplify milk-associated CCs in farm environments. Furthermore, cow udders may be contaminated by contact with their contaminated environment, and milk could get contaminated during the milking process^2,65^. *Lm* biofilms have even been detected in milking equipment in farms^66^, increasing the risk of milk contamination. Moreover, the better growth of hypervirulent clones at 37 °C in presence of salt, as compared with hypovirulent CCs (in line with the work of Horlbog et al.^67^), may account in part for the greater gut colonization capacity of hypervirulent CCs, as the gut lumen is known to contain high salt concentrations.

Many studies have shown that *Lm* may produce biofilm and/or be tolerant to commonly used disinfectants such as BC, leading to its persistence in food production environment during long periods of time^68^. More specifically, CC121, CC9, CC31, and CC13 have been shown to better tolerate BC^20,21,36^, whereas CC14 has been shown to produce biofilm more efficiently than CC121^21^. Whereas there are contradicting data in the literature regarding differences of biofilm formation capacities in *Lm* sub-populations^69^, our study shows that the hypovirulent clones CC9 and CC121 produce more biofilm and grow better than CC1, CC2, CC4, and CC6 in presence of low BC concentrations. At 4 °C, adjunction of BC stimulates biofilm production by CC4, CC9, and CC121 as compared with growth conditions without BC. These data strongly suggest that surface disinfection using BC may provide hypovirulent CCs with a survival and/or growth advantage, favoring their higher persistence on surfaces or equipment treated with BC and to their frequent transfer to new products in contact with contaminated surfaces. The high prevalence of CC9 and CC121 in products that need to be processed before consumption may therefore be a consequence of the use of BC for disinfection purposes on production facility surfaces. The fact that no growth was observed with 200 mg/L BC for any tested strain confirms that BC tolerance genes provide tolerance to low BC concentration only, which has been shown to occur after sanitation in meat-processing plants^56^.

The distribution of BC tolerance genes (*qac*, *bcrABC, emrC*, *emrE*, *qacC,* and *qacA*) in the isolates analyzed in this study fully correlates with their BC tolerance phenotype and their capacity to form biofilm in presence of BC. We confirmed the role of *qac* and *bcrABC* on BC tolerance in CC9 and CC121 isolates based on mutagenesis, complementation, and BC sensitivity assays^42,57,58^. By analyzing 2928 *Lm* genomes representative of the *Lm* isolates circulating in France, we could show that 32.1% of isolates may be able to persist in presence of low BC concentrations, and that BC tolerance genes are enriched in hypovirulent clones. The uneven proportion of isolates harboring BC tolerance genes and more generally stress resistance genes among CCs suggests that these have variable levels of adaptation to the food-processing environment, and that more-sensitive isolates could become tolerant/resistant through the acquisition of BC tolerance and stress resistance genes, as these are often present on mobile genetic elements^42,45,57–59^. In order to limit the spread of disinfectant-tolerant bacteria and their persistence in food production environments, the use of combined antimicrobials approaches has been proposed, as well as the use of non-chemical approaches^70–72^.

As compared with CC1 and CC4, CC2, and CC6 have lower proportions of clinical isolates and of isolates from dairy products (Fig. 1a and Fig. 2a), as well as higher proportions of isolates from meat products (Fig. 2b). In addition, higher proportions of CC2 and CC6 isolates harbor BC tolerance genes than CC1 and CC4 isolates (Supplementary Fig. 7). These observations suggest that CC2 and CC6 may have more diversified phenotypes, combining vertically transmitted features of host-adapted clones (i.e., CC1 and CC4) and horizontally transferred resistance to BC typical of clones highly adapted to the food production environment (i.e., CC9 and CC121).

In summary, our study shows three distinct patterns among major *Lm* clones: (i) clones that are host-associated, highly prevalent in dairy products, exhibiting a low adaptation to food production environments and rarely harboring BC tolerance genes (i.e., CC1 and CC4); (ii) clones with low adaptation to the host but persisting efficiently in food-production environment owing to efficient biofilm formation and tolerance to disinfectants due to high prevalence of BC tolerance genes (i.e., CC9 and CC121); and (iii) intermediary clones that may be in the process of transitioning from host-associated to saprophytic lifestyles through the loss of virulence^73^ and/or the acquisition of genes involved in tolerance to disinfectants (i.e., CC2 and CC6)^45^. Genes potentially involved in adaptation to the host or to a saprophytic lifestyle were identified in our study using a GWAS approach and it will be interesting to test their contribution to these lifestyles experimentally. The over-representation of genes involved in replication, recombination, and repair in hypovirulent CCs may indicate a higher exposure to genotoxic conditions, whereas the over-representation of genes involved in cell wall and membrane biogenesis in hypervirulent CCs may reflect the selection of genes involved in interactions with the host (see Supplementary Fig. 10).

The unveiling of the relative distribution of *Lm* lineages and CCs in different food categories also has strong implications for risk assessment in food processing plants and should be considered to identify and control food contamination routes and sources, as well as to guide public health authorities to better control the health hazards associated with *Lm*.

## Methods

### Strain collection used in this study

A total of 6641 isolates collected between January 2005 and May 2016 at the National Reference Center for *Listeria* (NRCL) in the context of the French surveillance of listeriosis were used for investigation of CC distribution in food sources (food isolates, *n* = 3333; clinical isolates, *n* = 3308). Similar to Maury, Tsai et al.^15^, clinical isolates were collected with a high level of exhaustiveness owing to mandatory declaration of listeriosis in France and are therefore highly representative of the listeriosis cases that occurred in France during the study period. Regarding the food isolates, 2623 (78.7%) were collected in the context of food alerts, which are triggered when contaminated food products are on the market. The remaining 710 isolates (21.3%) were collected in the context of own-checks performed by food industries or in case of investigations following neurolisteriosis cases. Therefore, they largely represent the *Lm* isolates circulating in France, to which the population is exposed.

All isolates received at the NRCL before 2015 were routinely typed by Pulse-Field Gel Electrophoresis (PFGE) according to the PulseNet standardized procedures with AscI and ApaI enzymes^74^, from which the MLST clonal complexes can be deduced^15^, and all isolates received after 2015 were genotyped by core genome MLST (cgMLST)^10,22^ (*cf*. methods below).

This collection of isolates was deduplicated in order to avoid any bias in the analyses. More specifically, only one isolate was considered in case of MN listeriosis (mother’s isolates were kept). Regarding the food isolates, only one was considered when several had identical cgMLST type or CC (*cf*. methods below) and identical food alert number or precise food product.

### Genome sequencing, typing, and genome analyzes

DNA extraction, whole-genome sequencing and cgMLST were routinely performed in the context of the French surveillance of listeriosis with the same procedures than in Moura et al.^22^. In brief, Genomic DNA was extracted using a DNeasy Blood and Tissue Extraction kit (Qiagen, Denmark) and used for whole-genome sequencing on an Illumina NextSeq 500 (2 × 150 bp) platform (Illumina, CA, USA). Reads were trimmed to eliminate adapter sequences and discard reads with Phred scores ≤ 20. De novo assembly of Illumina reads was performed using CLC Assembly Cell version 4.3.0 (QIAGEN, Venlo, Netherlands) or SPADES version 3.11.0^75^. MLST CCs^9^ were deduced from genome sequences, when available, using the BIGSdb platform (http://bigsdb.pasteur.fr/listeria). In case, only PFGE profiles were available, CCs were deduced using the PFGE/MLST library described in Maury, Tsai et al.^15^. Only CCs identified with high confidence were considered (SST ≥ 97.5% and DST ≥ 1%)^15^.

A total of 2928 genomes of non-redundant *Lm* isolates collected from 2015 to 2018 in the context of the French surveillance was analyzed for the presence/absence of BC tolerance genes (*qac* (Tn6188)^38,39^, *bcrABC*^42,57,58^*, emrE*^44^, *emrC*^45^, *qacC*^47,48^, and *qacA*^46^) as well as stress resistance genes^21^, which are not part of the *Lm* core genome^10,15^ using BLASTN (BLAST+ v. 2.6.0; min identity: 80%; min alignment: 80%; Blastn word size: 11).

### Gene association with dairy or meat origins

Genomes were annotated using Prokka version 1.12^76^ with the default parameters for Gram-positive bacteria. Pangenome of the 1129 genomes from isolates collected from dairy and meat samples was performed using Roary version 3.6^77^ with a minimum identity of 90%. Identification of dairy- and meat-associated genes was performed using Scoary version 1.6.10^78^ using default parameters. Only genes showing a Bonferroni corrected *p* value higher than 0.05 were considered, among which those with odd ratio higher than 1 were considered as associated to a dairy or meat origin, depending on the origin tested. Genes associated with dairy or meat origins were classified into COG using the online eggNOG-mapper tool version 4.5.1^79^ (http://eggnogdb.embl.de).

### Isolates and culture conditions

Among the NRCL collection of isolates, 42 isolates were selected to represent the *Lm* phylogenetic diversity of each clone of interest (CC1, CC2, CC4, CC6, CC9, and CC121; seven isolates per CC), based on their cgMLST profiles (Supplementary Data 2). In brief, we performed a single linkage cluster analysis of all sequenced isolates based on their cgMLST profiles using Bionumerics version 7.6.2 (bioMérieux, Marcy l’Etoile, France), and we selected one isolate per major clade of each CC. Strains were stored at − 80 °C in Cryobank (Mast Group Ltd, Bootle, UK) and revived by plating onto Columbia agar (bioMérieux, Marcy l’Etoile, France) and grown overnight at 37 °C. Then, single colonies were grown in BHI broth (growth and colonization experiments) or MCDB 202 broth (MyBioSource, San Diego, CA, USA) supplemented with 1% yeast extract (Becton Dickinson, USA) and glucose 3.6 g/L (Sigma-Aldrich, USA), adjusted to pH 7.3 and filtered (for assessment of biofilm production efficiency).

### Effect of pH, temperature, NaCl, and BC on growth

Microbial growth efficiency was assessed as described previously in Maury et al.^73^. In brief, bacteria were first cultured overnight on BHI agar at 22 °C or 37 °C, and one colony was used to inoculate 5 mL of BHI broth. For growth in varying pH, temperatures and NaCl concentrations, stationary-phase cultures were diluted in BHI supplemented or not with NaCl (0.09 or 1.1 M, final concentrations) and adjusted to pH 5, 6 or 7, and grown at 22 °C or 37 °C until OD_600nm_ 0.1. For growth in presence of BC, stationary-phase cultures were diluted in modified MCDB 202 supplemented or not with varying BC concentrations (1.5, 4.5, 7, 10, or 200 mg/L) (Sigma-Aldrich; Saint. Louis, MO, USA). Two hundred µl of the diluted cultures were then transferred into Bioscreen C 100-well plates (Oy Growth Curves Ab Ltd., Helsinki, Finland) for microbial growth monitoring at 22 °C or 37 °C by measuring the OD_600nm_ every 15 min with shaking until stationary phase (during at least 15 h). Growth at 4 °C was also tested in BHI by adding 200 µl of culture to wells of 96 tissue culture test well plates TPP (Dutsher, Brumath, France). Plates were incubated at 4 °C in a refrigerated room with shaking and the OD_600nm_ was recorded every day during 9 days. For all conditions, OD_600nm_ values of non-inoculated wells (blanks) were subtracted from those of inoculated ones to compensate for the background noise. Each strain was tested in triplicate for each condition. Mean OD_600nm_ values per strain were used to calculate areas under the curves over time using the auc function of the AUC R package.

### Effect of temperature and BC on biofilm

Methods for assessing biofilm formation efficiency were adapted from Chavant et al.^80^, Pan et al.^81^. Combrouse et al.^82^. Overnight cultures were grown at 37 °C in modified MCDB 202 (*cf*. methods above) and centrifuged at 7000 rpm during 5 min. Bacteria were re-suspended in modified MCDB 202 (see above) without or with varying BC concentrations (1.5, 4.5, 7, or 10 mg/L) to reach OD_600nm_ 0.1, and 300 µl of this suspension was transferred in wells of 96-well polystyrene microplates (Nunclon Delta 96-well MicroWell Plates, Thermo Fischer Scientific, Waltham, MA, USA), in triplicate. Microplates were incubated at 37 °C during 48h or at 4 °C during 20 days. Wells were washed gently twice with 200 µl of sterile distilled water, 200 µl of a violet crystal solution 1% (Química Clínica Aplicada S.A, Tarragona, Spain) was added and plates were incubated 45 min in the dark at room temperature. Violet crystal was removed, wells were washed twice with 200 µl of sterile distilled water and 200 µl of ethanol 95% was added to detach the colored biofilm. The resulting colored suspension was transferred in 96-well microplates (Tissue Culture Test Plate, TPP) and quantified by measuring the OD_595nm_ using a Multiskan plus plate reader (Thermo Fisher Scientific, Waltham, MA, USA). Average OD_595nm_ from un-inoculated wells containing modified MCDB 202 was subtracted from the OD_595nm_ of test wells, and mean OD_600nm_ values per strain were determined.

### Mutagenesis of *qac* (Tn6188) and *bcrABC*

The *qac* gene and its flanking regions were PCR amplified using oligonucleotide primers *Bam*HI-*qac*fwd (5′-GGGGATCCTGCAACAATCGCTCCCGTTA-3′) and *Sal*I-*qac*rev (5′-GGGTCGACAGTAATTGCTGGACCCTGCC-3′). The fragment was then purified and digested with *Bam*HI and *Sal*I and cloned into the pLR16-PheS shuttle vector (kind gift from professor Anat Herskovits, Tel Aviv University), resulting in the recombinant plasmid pLR16-*qac*. An in-frame deletion was introduced in the *qac* gene by PCR using oligonucleotide primers Δ*qac*fwd (phosphoryl-5′-ATTGTTATTCGCCCTCCT-3′) and Δ*qac*rev (phosphoryl-5′-TCTTTCTTCCGCAAACCT-3′). The resulting recombinant plasmid pLR16-Δ*qac* was shuttled into *Lm* isolate CLIP 2016/00360 via conjugation from *Escherichia coli* S17–1. Mutagenesis was then performed as previously described^83^.

The *bcrABC* gene cluster was PCR amplified using the oligonucleotide primer pairs *Sac*I-*bcrABC*fwd (5′-GGGAGCTCGATTCTGGAACATCCCTATC-3′)–*Sal*I-*bcrABC*rev (5′-GGGTCGACGTATAATCCGGATGCTGCCC-3′) and *Eag*I-*bcrABC*fwd (5′-GGCGGCCGGATTCTGGAACATCCCTATC-3′)–*Sal*I-*bcrABC*rev (5′-GGGTCGACGTATAATCCGGATGCTGCCC-3′). The fragments were then purified and digested with *Sac*I, *Eag*I, and *Sal*I and cloned into pPL2 shuttle vector, resulting in recombinant plasmid pPL2-*bcrABC*. The plasmid was then transferred into *Lm* CLIP 2009/00521 via conjugation from *E. coli* S17–1. Plasmid integration was verified by PCR using oligonucleotide primers NC16 (5′-GTCAAAACATACGCTCTTATC-3′) and PL95 (5′-CACATAATCAGTCCAAAGTAGATGC-3′) as previously described^84^.

### BC sensitivity assessment of ∆*qac* and pPL2::*bcrABC* strains

*Lm* strains to be tested (CLIP 2009/00521+pPL2::*bcrABC*, CLIP 2009/00521WT, CLIP 2016/00360∆*qac,* and CLIP 2016/00360WT) were grown in BHI overnight at 37 °C with shaking. Cultures were diluted 20-fold in BHI containing BC or not (0 mg/L–10 mg/L) and dilutions were incubated at 37 °C with shaking for 2 h. Sensitivity of the WT and mutant strains was evaluated by measuring the OD_600nm_ of all cultures. Three independent experiments were performed.

### Colonization

We used 7- to 8-week-old mEcad E16P KI female mice in a C57BL/6J genetic background^62^. For colonization experiments, a minimum of four mice were used for each tested CC. Mice had their food restricted overnight with free access to water before inoculation. *Lm* inoculum was prepared by diluting overnight pre-cultures to the 1:20 in BHI broth and incubated at 37 °C until they reach 8 × 10^8^ bacteria/mL (~ OD_600nm_ 0.8). Bacteria were washed in phosphate-buffered saline (PBS) and strains belonging to the same CC were mixed together equiproportionally. Mice were orally inoculated with 2 ×  10^7^ of total bacteria and 300 µl of CaCO_3_ (50 mg/mL) and placed in separated cages for convenient collection of total stools per mice every day during 10 days. Total stools from each mice were homogenized in PBS and 10^0^ to 10^−7^ dilutions in PBS were plated on ALOA agar plates (bioMérieux, Marcy l’Etoile, France). CFU enumerations were performed after 48h of incubation at 37 °C.

In order to evaluate infection of deeper organs, a similar protocol was used, except that each mouse was infected with 2 × 10^8^ bacteria and that total stools per cage were collected every day during 4 days. Then total stools were homogenized in PBS and 10^0^ to 10^−7^ dilutions in PBS were plated on ALOA agar plates (bioMérieux, Marcy l’Etoile, France). CFU enumerations were performed after 48h of incubation at 37 °C. In addition, a stool sample was collected from each mouse every day for evaluation of bacteria concentration per mouse. CFUs per day and per mouse were deduced from the total CFU counts per cage and CFU concentration per mouse. Mice were killed on the 4th day for CFU enumeration in organs. Organs were homogenized in PBS. Before homogenization, small intestine, cecum and colon were rinsed with DMEM and incubated for 2 h at room temperature in DMEM supplemented with gentamicin (100 µg/mL; Sigma-Aldrich). All the procedures used in this study are in agreement with the guidelines of the European Commission for the handling of laboratory animals, directive 86/609/EEC. They were approved by the ethical committee of Institut Pasteur (CETEA-C2EA no. 89) under the number dap170057 and received an agreement from the ministry of higher education, research, and innovation under the number APAFIS#14644–2018041116183944 v1.

### Statistics and data analyzes

As in Maury, Tsai et al.^15^, association of CCs with food origins (dairy products, meat products, and seafood products) was tested using *χ*^2^ tests. Sequential Bonferroni correction was used to adapt significance thresholds according to the number of tests performed^85^. In order to quantify the strength of association between the frequency of clones in dairy and meat products and their clinical frequency, linear regressions were performed using the lm function implemented in the basic R distribution. To avoid any bias owing to rare clones, weights were applied to take into account the total number of isolates in each clone.

The Wilcoxon rank sum test (equivalent to Mann–Whitney *U* test) was used to compare clones or groups of clones (hypervirulent versus hypovirulent clones) pairwise regarding their capacity to form biofilm (OD_595nm_), grow in different conditions (AUC), colonize the gut of mice (AUC) and infect mice organs using the pairwise.wilcox.test function with option paired = FALSE available in the basic R distribution. The Holm correction was used in order to adjust p-values according to the number of tests performed. When ex-aequo values of AUC or OD_595nm_ were obtained, the nparcomp function that performs non-parametric tests for pairwise comparisons, implemented in the nparcomp R package, was used with the option type = Tukey.

### Reporting summary

Further information on research design is available in the Nature Research Reporting Summary linked to this article.

## Supplementary information

Supplementary Information

Description of Additional Supplementary Files

Supplementary Data 1

Supplementary Data 2

Supplementary Data 3

Supplementary Data 4

Reporting Summary

Source Data

## Data Availability

The authors declare that all the data supporting the findings of this study are available within the article and its supplementary information files. Data underlying Fig. 4 and 6 as well as Supplementary Figs. 3, 4, 5, 6, 8, and 9 are provided in a Source Data file. Genome data analyzed in this study were generated in the context of the epidemiological surveillance of listeriosis in France. As mentioned in the “Décret no. 2016–806 du 16 juin 2016 relatif aux centres nationaux de reference pour la lute contre les maladies transmissibles” and in the “Arrêté du 16 juin 2016 fixant le cahier des charges des centres nationaux de références pour la lute contre les maladies transmissibles”, all samples collected at the NRCL as well as the data generated from these samples belong to the French Government and constitute a “national collection of biological resources of interest to public health”, which has to remain under medical and industrial confidentiality, and therefore they cannot be made publicly available”.
