# Author Correction: Hypervirulent *Listeria monocytogenes* clones’ adaptation to mammalian gut accounts for their association with dairy products

**DOI:** 10.1038/s41467-019-11625-8

**Published:** 2019-08-06

**Authors:** Mylène M. Maury, Hélène Bracq-Dieye, Lei Huang, Guillaume Vales, Morgane Lavina, Pierre Thouvenot, Olivier Disson, Alexandre Leclercq, Sylvain Brisse, Marc Lecuit

**Affiliations:** 1 Biology of Infection Unit, Inserm U1117, Institut Pasteur, 75015 Paris, France; 2 National Reference Centre and WHO Collaborating Centre for Listeria, Institut Pasteur, 75015 Paris, France; 3 Microbial Evolutionary Genomics Unit, CNRS UMR 3525, Institut Pasteur, 75015 Paris, France; 4 Université Paris Diderot, Université de Paris, 75013 Paris, France; 5 Paris Descartes University, Institut Imagine, Necker-Enfants Malades University Hospital, Division of Infectious Diseases and Tropical Medicine, APHP, 75006 Paris, France; 6 Present Address: Biodiversity and Epidemiology of Bacterial Pathogens Unit, Institut Pasteur, 75015 Paris, France

**Keywords:** Microbial ecology, Bacterial genetics, Pathogens

Correction to: *Nature Communications* 10.1038/s41467-019-10380-0, published online 06 June 2019

The original version of this Article contained an error in the spelling of the word “adaptation” both in the Title and Abstract, which was incorrectly written as “adaption”. This has been corrected in both the PDF and HTML versions of the Article.

