# Supplementary material for: Hypervirulent Listeria monocytogenes clones’ adaption to mammalian gut accounts for their association with dairy products
Source: Nat Commun. 2019 Jun 6;10:2488. doi: 10.1038/s41467-019-10380-0 (PMC6554400; doi:10.1038/s41467-019-10380-0)
Supplement: Supplementary file 1 — Supplementary Information [file 41467_2019_10380_MOESM1_ESM.pdf]

Supplementary Information

Hypervirulent *Listeria monocytogenes* clones' adaption to mammalian gut accounts for their association with dairy products

Maury et al.

Supplementary Figures

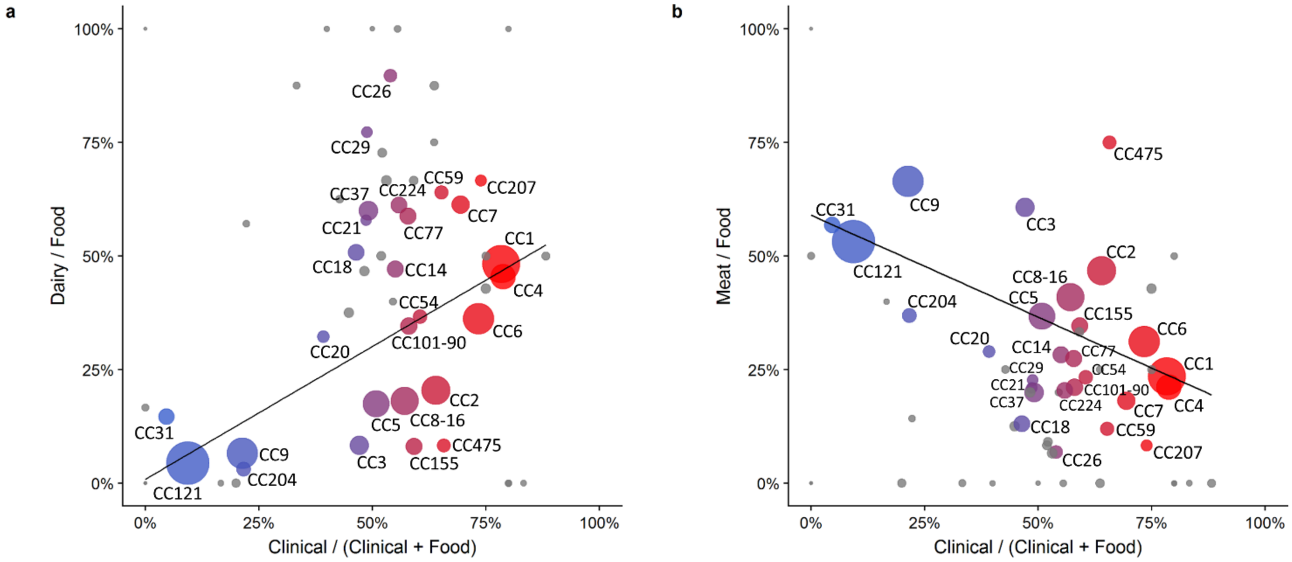

Supplementary Figure 1: Relationship between clone frequency in dairy and meat products versus clinical frequency

Frequency of clones in dairy products (**a**) and meat products (**b**) as a function of clinical frequency. Major CCs representing individually at least 0.5% of all the isolates of the study are shown with similar colors than in Fig. 1a and are indicated next to the corresponding dots. The other CCs, considered as minor, are represented in grey. Linear regressions of isolate frequency in dairy products (**a**; $p < 1.10^{-7}$, $R^2 = 0.425$) and in meat products (**b**; $p < 1.10^{-7}$ and $R^2 = 0.426$) against clinical frequency were weighted by number of isolates per CC.

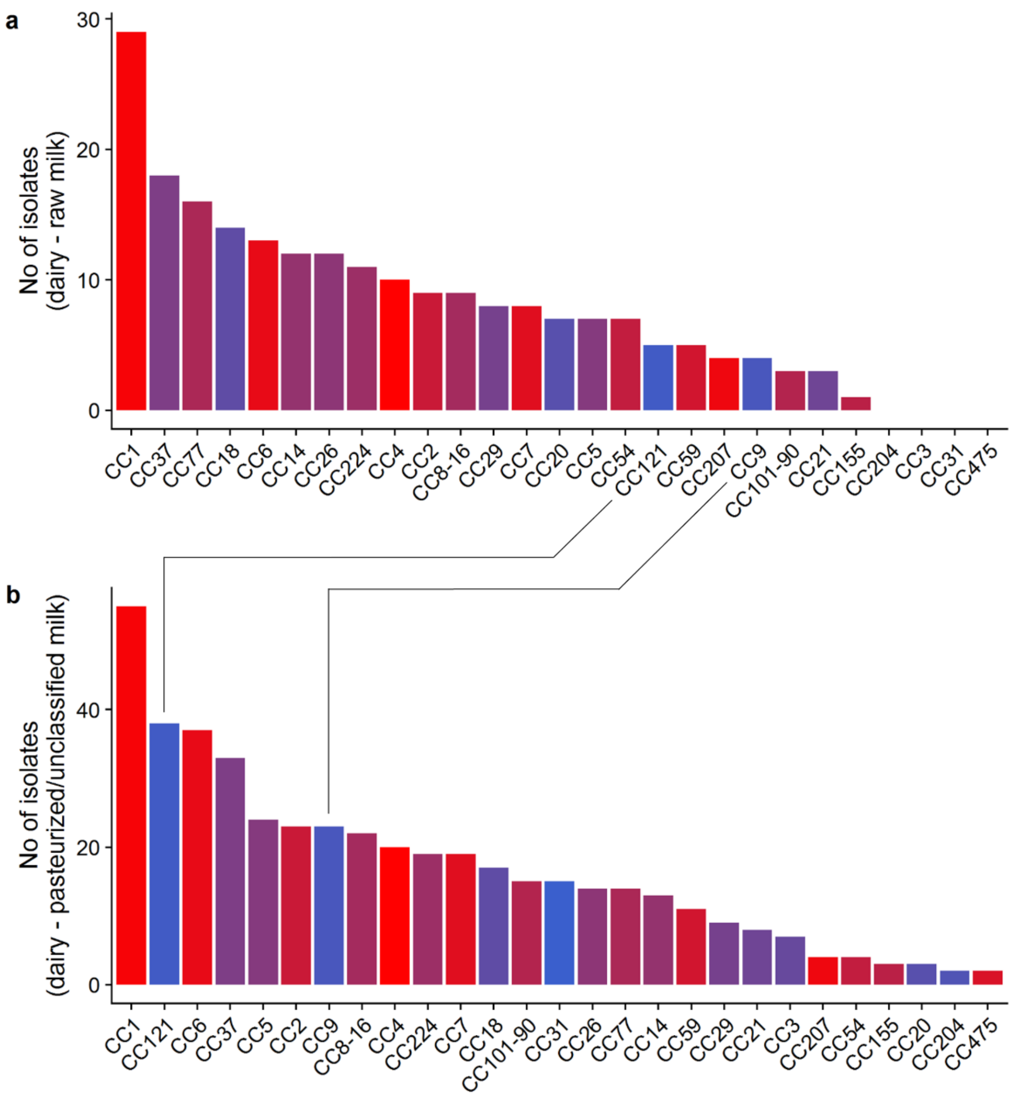

Supplementary Figure 2: Number of isolates per clone in row and pasteurized milk-derived products

All isolates collected from dairy products are shown (n = 758). Major CCs representing individually at least 0.5% of all the isolates of the study are shown with similar colors than in Fig. 1a. **a** Number of isolates collected from raw milk-derived products. **b** Number of isolates collected from dairy products made of pasteurized or unknown type of milk. Clones are ordered by number of isolates of each type of dairy product. Isolates were considered to be isolated from dairy products made of raw milk only if it was clearly indicated as such, if not, dairy products were considered as made of pasteurized or unknown type of milk. Black lines between panels **a** and **b** highlight a shift of CC9 and CC121 between the two types of dairy products.

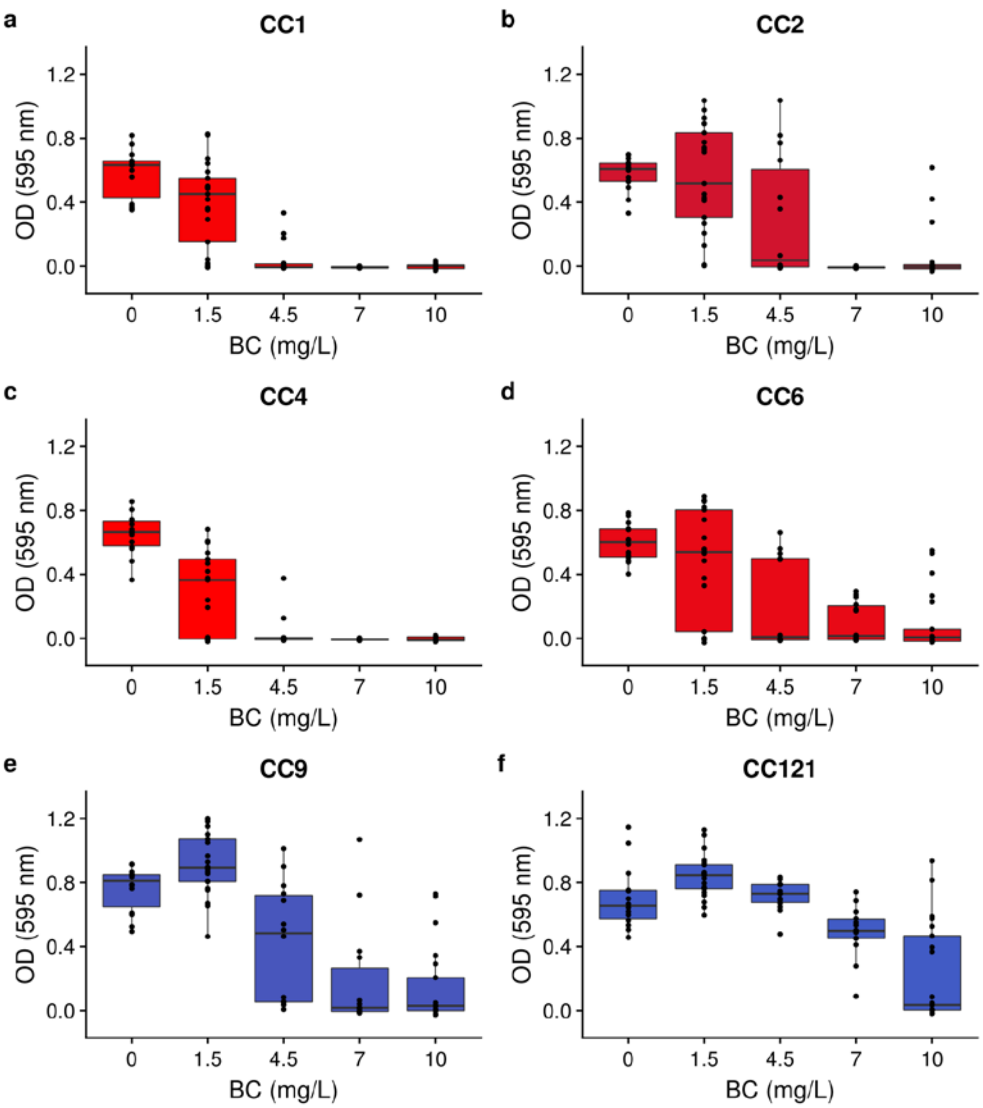

Supplementary Figure 3: Effect of increasing BC concentrations on biofilm formation per clone

Biofilm formation capacity in absence or presence of 1.5, 4.5, 7 and 10 mg/L of benzalkonium chloride (BC) at 37 °C is shown for 42 isolates representative of CC1, CC2, CC4, CC6, CC9 and CC121 (seven isolates per CC). Biofilm quantification was performed after 48h of static growth in modified MCDB 202 medium with or without BC using a violet crystal labeling-based method (see Methods section) and is shown as OD_{595nm}. Colors are as in Fig. 1a. In boxplots, the boxes delimit the first (25%) and third (75%) quartiles while the bars delimit the second quartile (median). Outliers are outside the whiskers. n = seven strains per clone tested three times independently for each condition. BC: Benzalkonium chloride. Source data are provided as a Source Data file.

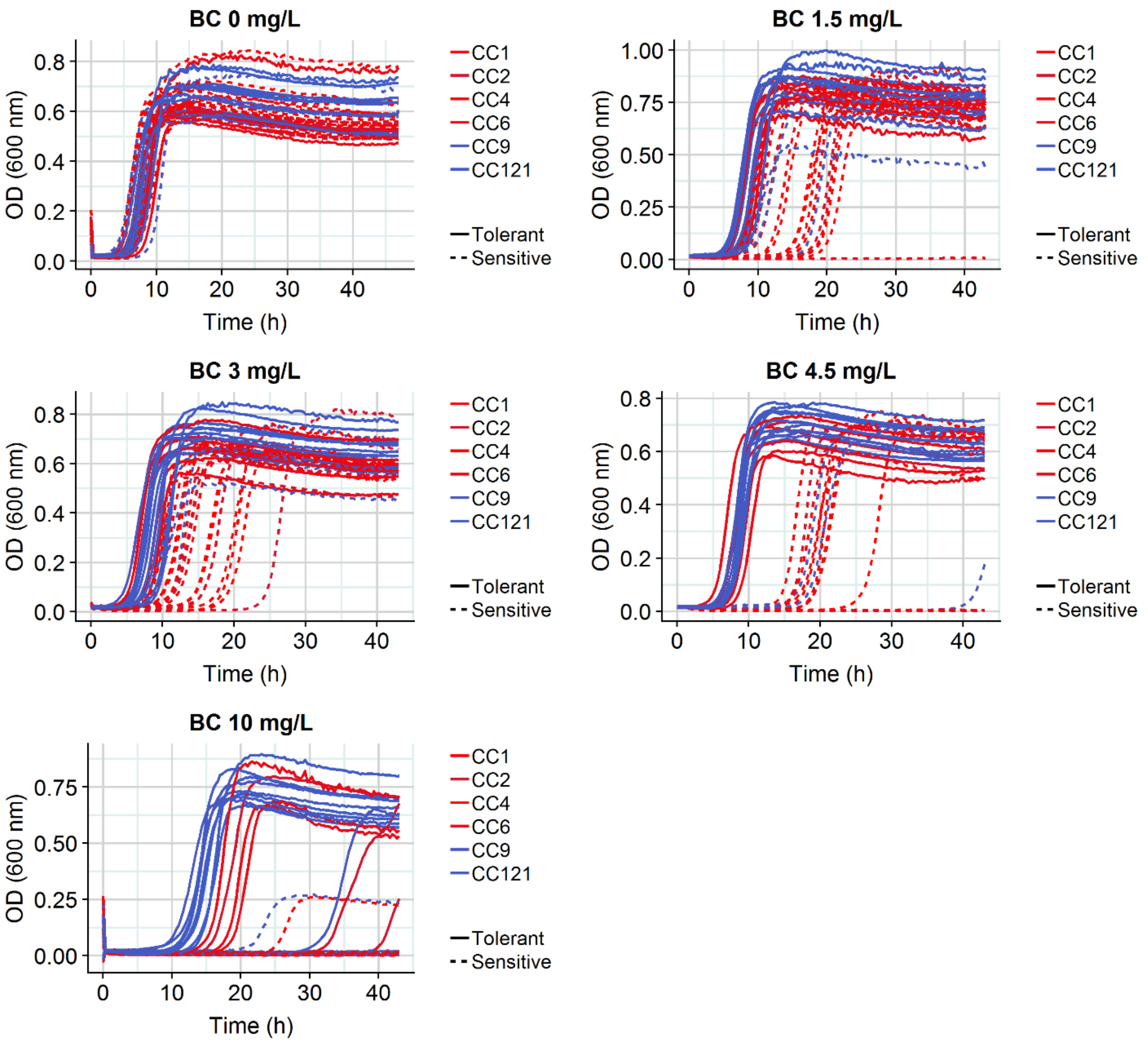

Supplementary Figure 4: Effect of increasing BC concentrations on growth

Growth in BHI broth at 37 °C in absence or presence of 1.5, 3, 4.5 or 10 mg/L of benzalkonium chloride (BC) was assessed for 42 representative isolates of CC1, CC2, CC4, CC6, CC9 and CC121 (seven isolates per CC). Growth was evaluated by measuring the OD_{600nm} every 15 min during 43 h. BC-tolerant (solid lines) and -sensitive (dashed lines) isolates are indicated. Colors are as in Fig. 1a. BC: Benzalkonium chloride. Source data are provided as a Source Data file.

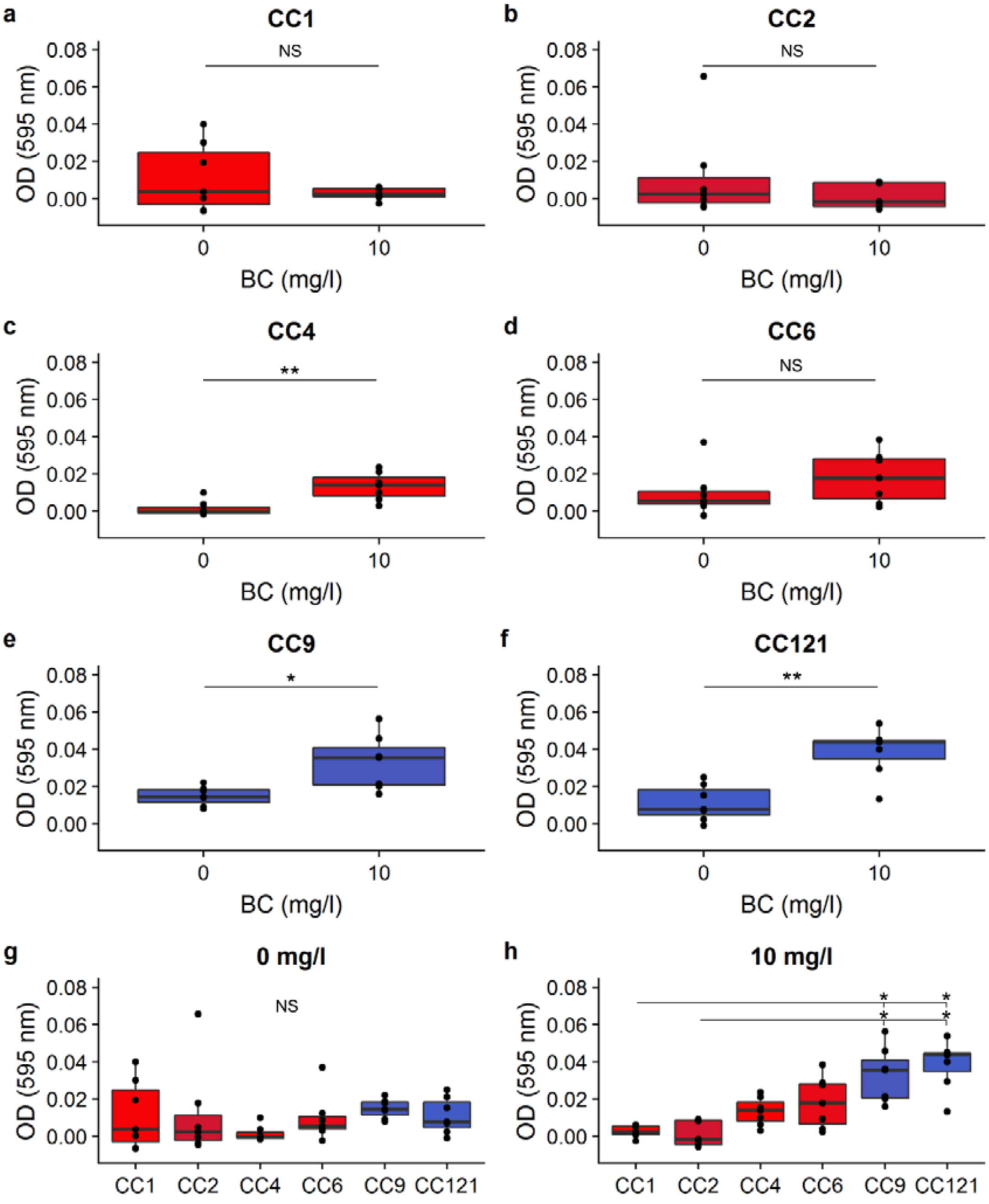

Supplementary Figure 5: Effect of BC on biofilm formation at 4 °C

Biofilm formation capacity in absence or presence of 10 mg/L of benzalkonium chloride (BC) at 4 °C is shown for 42 isolates representative of CC1, CC2, CC4, CC6, CC9 and CC121 (seven

isolates per CC). Biofilm quantification was performed after 48 h of static growth in modified MCDB 202 medium with or without BC using a violet crystal labeling-based method (see Methods section) and is shown as OD_{595nm}. **a-f** Biofilm formation capacity in absence and presence of 10 mg/L of BC per clone. **g, h** Comparison of biofilm formation capacities between clones in absence (**g**) and presence of 10 mg/L of BC (**h**). Colors are as in Fig. 1a. In boxplots, the boxes delimit the first (25%) and third (75%) quartiles while the bars delimit the second quartile (median). Outliers are outside the whiskers. Statistics were performed using the Mann-Whitney U test with holm's correction. **: $p < 0.01$; *: $p < 0.05$. NS: Not significant. BC: Benzalkonium chloride. Source data are provided as a Source Data file.

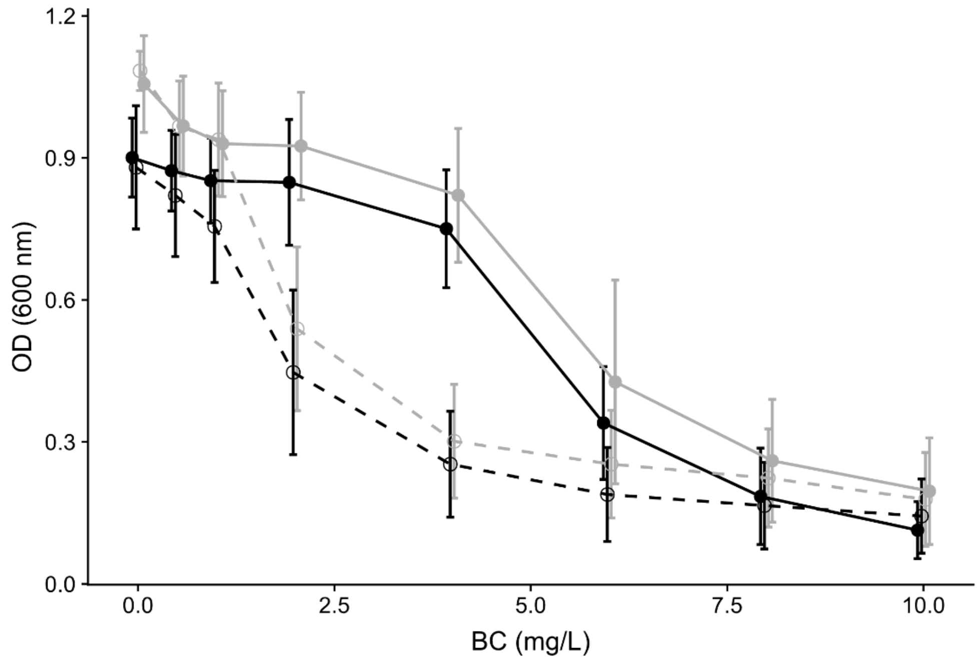

Supplementary Figure 6: Effect of *qac* (Tn6188) and *bcrABC* gene cassette on BC tolerance of CC9 and CC121

In order to validate the role of *qac* and *bcrABC* in BC tolerance of CC9 and CC121 isolates, we constructed the mutant strain CLIP 2016/00360Δ*qac* (CC121 strain where *qac* has been removed) and the complemented strain CLIP 2009/00521+pPL2::*bcrABC* (CC9 strain where a pPL2 plasmid harboring the *bcrABC* gene cassette under its native promoter has been inserted intra-chromosomally). We then compared these strains with their corresponding WT strains by measuring the OD_{600nm} after 2 h of growth at 37 °C in BHI with various BC concentrations (from 0 to 10 mg/L). The graph shows the OD_{600nm} for all tested BC concentrations. Error bars show the standard error of the mean, taking into account three independent experiments. Black solid line: CLIP 2009/00521+pPL2::*bcrABC*, black dashed line: CLIP 2009/00521WT, grey dashed line: CLIP 2016/00360Δ*qac*, grey solid line: CLIP 2016/00360WT. (i.e. grey: CLIP 2016/00360-deriving strains; black: CLIP 2009/00521-deriving strains; dashed lines: strains with no BC tolerance gene; solid lines: strains with either *qac* or *bcrABC*). BC: Benzalkonium chloride. Source data are provided as a Source Data file.

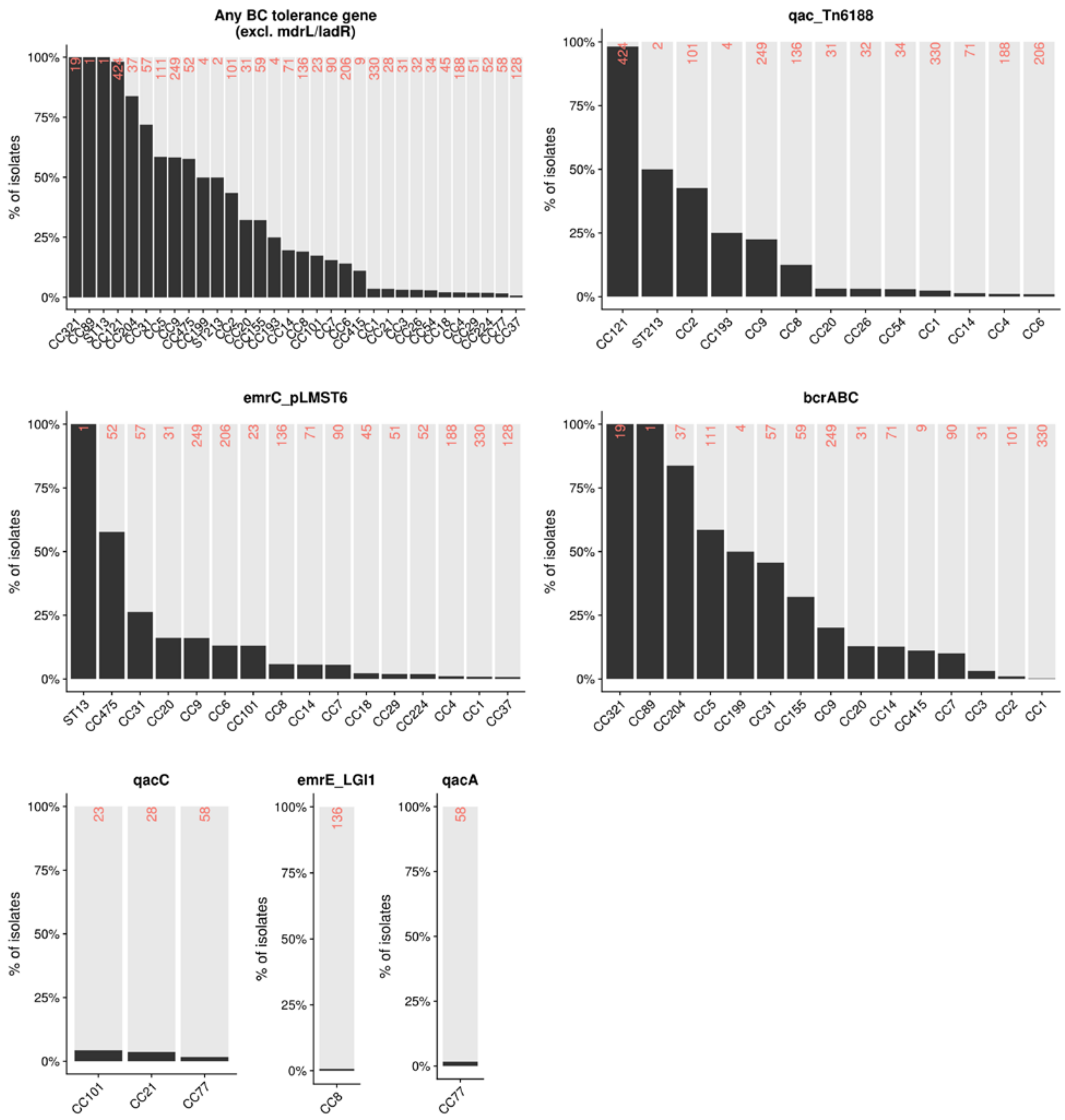

Supplementary Figure 7: Prevalence of BC tolerance genes in *Lm* clones

We analyzed a set of 2,928 genome sequences of non-redundant *Lm* isolates collected from 2015 to 2018 in the context of the French surveillance of listeriosis. Percentages of isolates harboring BC-tolerance genes (i.e. *qac*, *bcrABC*, *emrC*, *qacC*, *emrE* and *qacA*) per clone are shown. The graphic on the top-left shows the number of isolates harboring at least one BC tolerance gene cited

above. *malR* and *ladR* were found in almost all isolates, as they are part of the *Lm* core genome[15,10]. Black: gene present. Grey: gene absent. Total numbers of isolates per clone are indicated on top of each bar. Only clones with at least one isolate harboring the considered BC-tolerance gene are shown in each plot. *bcrABC* was considered present only if all genes of the cassette were present.

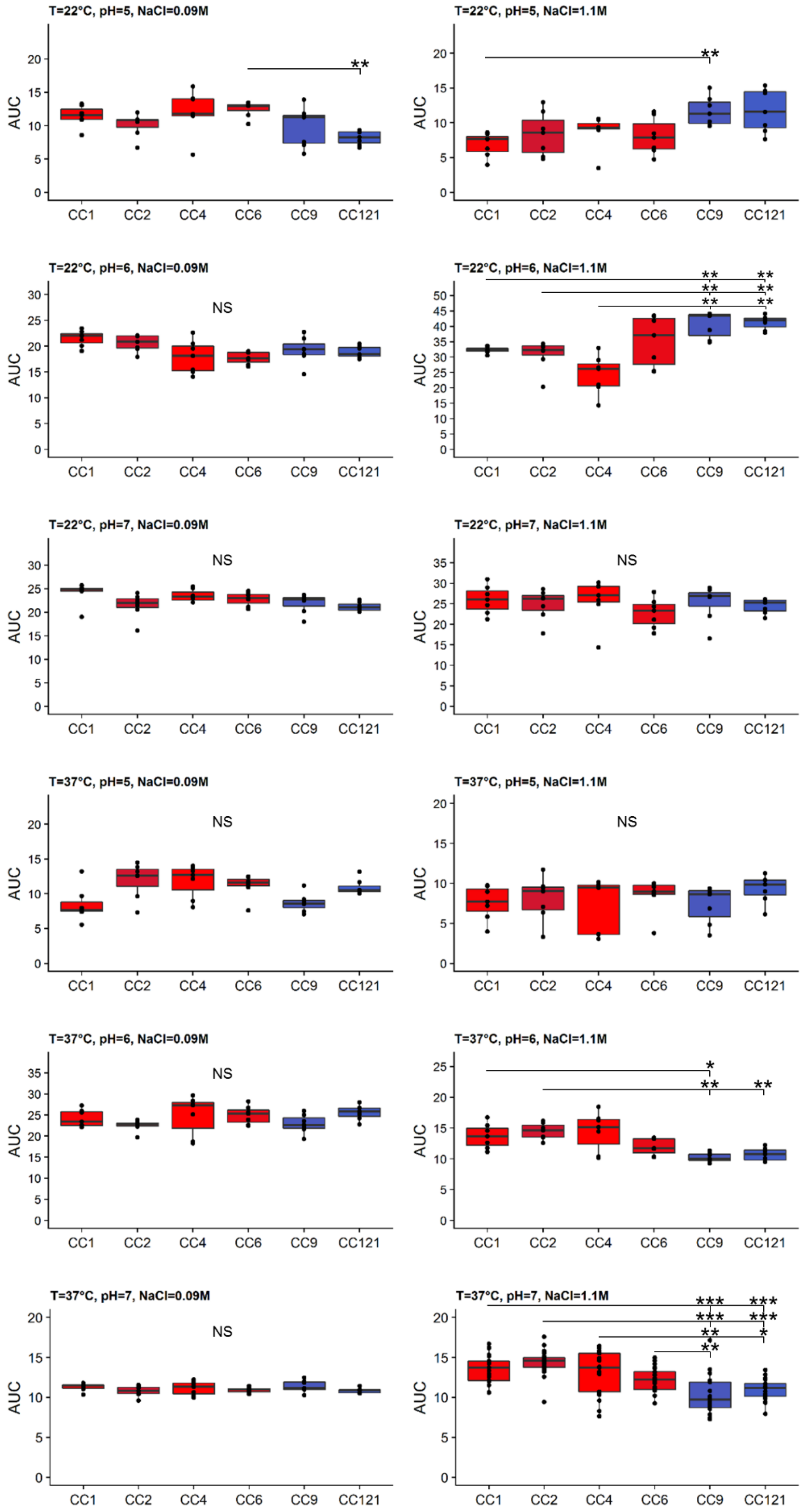

T=22°C, pH=5, NaCl=0.09M
T=22°C, pH=5, NaCl=1.1M
T=22°C, pH=6, NaCl=0.09M
NS
T=22°C, pH=6, NaCl=1.1M
T=22°C, pH=7, NaCl=0.09M
NS
T=22°C, pH=7, NaCl=1.1M
NS
T=37°C, pH=5, NaCl=0.09M
NS
T=37°C, pH=5, NaCl=1.1M
NS
T=37°C, pH=6, NaCl=0.09M
NS
T=37°C, pH=6, NaCl=1.1M
T=37°C, pH=7, NaCl=0.09M
NS
T=37°C, pH=7, NaCl=1.1M
AUC
CC1
CC2
CC4
CC6
CC9
CC121

Supplementary Figure 8: Effect of temperature, pH and salt on growth of *Lm* clones

Growth in BHI broth at 22 and 37 °C, pH 5, 6 and 7, in presence of 0.09 M or 1.1 M of NaCl is shown for 42 representative isolates of CC1, CC2, CC4, CC6, CC9 and CC121 (seven isolates per CC). Growth was evaluated by measuring the OD_{600nm} every 15 min during a minimum of 14 h. Areas under the growth curves shown in Supplementary Fig. 11 are shown per CC. Colors are as in Fig. 1a. In boxplots, the boxes delimit the first (25%) and third (75%) quartiles while the bars delimit the second quartile (median). Outliers are outside the whiskers. Statistics were performed using the Mann-Whitney U test with holm's correction. ***: $p < 10^{-3}$; **: $p < 10^{-2}$; *: $p < 0.05$. NS: Not significant. n = seven strains per clone tested three times independently. AUC: Area under the curve. Source data are provided as a Source Data file.

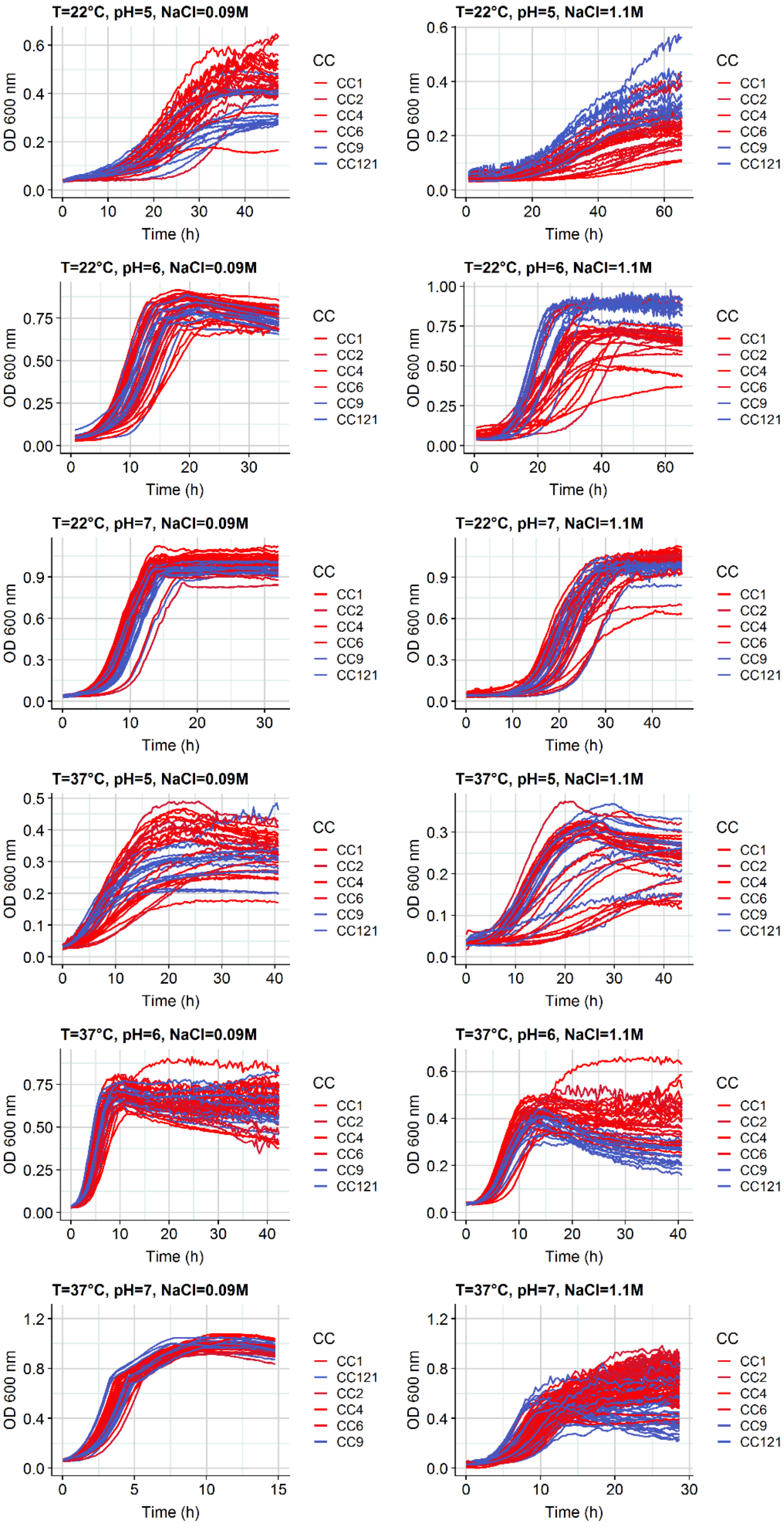

T=22°C, pH=5, NaCl=0.09M
T=22°C, pH=5, NaCl=1.1M
T=22°C, pH=6, NaCl=0.09M
T=22°C, pH=6, NaCl=1.1M
T=22°C, pH=7, NaCl=0.09M
T=22°C, pH=7, NaCl=1.1M
T=37°C, pH=5, NaCl=0.09M
T=37°C, pH=5, NaCl=1.1M
T=37°C, pH=6, NaCl=0.09M
T=37°C, pH=6, NaCl=1.1M
T=37°C, pH=7, NaCl=0.09M
T=37°C, pH=7, NaCl=1.1M
OD 600 nm
Time (h)
CC
CC1
CC2
CC4
CC6
CC9
CC121

Supplementary Figure 9: Effect of temperature, pH and salt on growth of *Lm* clones

Growth in BHI broth at 22 and 37 °C, pH 5, 6 and 7, in presence of 0.09 M or 1.1 M of NaCl is shown for 42 representative isolates of CC1, CC2, CC4, CC6, CC9 and CC121 (seven isolates per CC). Growth was evaluated by measuring the OD_{600nm} every 15 min during the indicated amount of time. Colors are as in Fig. 1a. Source data are provided as a Source Data file.

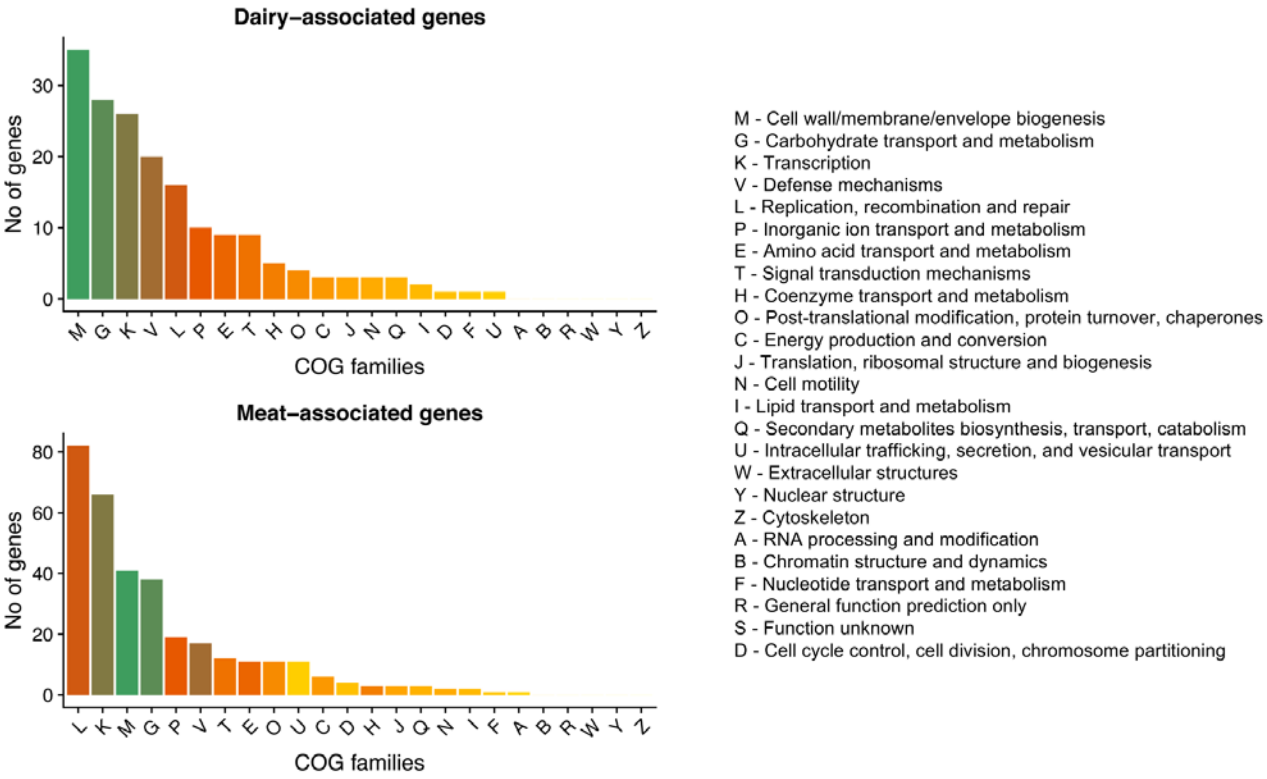

Supplementary Figure 10: Classification of genes associated with dairy and meat origins into clusters of orthologous groups

A genome wide association study was performed in order to identify genes significantly associated with dairy or meat origins among a set of 21,546 gene families of a pangenome built from 1,129 genomes. We obtained 455 and 916 gene families significantly associated with dairy and meat origins, respectively. The graphs show the classification of each of these genes into clusters of orthologous groups (COG). COG families are ordered per number of genes in each graph. Color gradient is set according to the order of the COG families in dairy-associated genes. Identical colors are used for each COG family in the graph of meat-associated genes. Top-left: COG families of dairy-associated genes. Bottom-left: COG families of meat-associated genes. Right: group of functions of each COG families.
