# Supplementary material for: Hypervirulent Listeria monocytogenes clones’ adaption to mammalian gut accounts for their association with dairy products
Source: Nat Commun. 2019 Jun 6;10:2488. doi: 10.1038/s41467-019-10380-0 (PMC6554400; doi:10.1038/s41467-019-10380-0)
Supplement: Supplementary file 2 — Description of Additional Supplementary Files [file 41467_2019_10380_MOESM2_ESM.docx]

**Description of Additional Supplementary Files**

**File Name: Supplementary Data 1**

**Description:** Number of isolates per source of isolation included in the study Distribution in food and clinical sources of 6,641 non-redundant Lm isolates collected between January 2005 and May 2016 by the National Reference Centre for Listeria in the context of the epidemiological surveillance of listeriosis in France. Number of isolates in each origin per CC is indicated, followed, between brackets, by the percentage of isolates relatively to the considered CC, and by the percentage of isolates relatively to the total number of isolates from the considered source. In the second part of the table, the percentage of food and clinical isolates per clone are calculated by dividing the number of isolates in food or clinical origins by the total number of isolates of the clone. In the third part of the table, the percentages of isolates in each food category per clone are calculated by dividing the number of isolates in the considered food category by the total number of food isolates of the clone. Clones are sorted by total number of isolates. Major CCs representing individually at least 0.5% of all the isolates of the study are shown above the horizontal line, representing in total 93.6% of all isolates (CCs shown in Figure 1). Distinctive associations of the major clones (above the horizontal line) with dairy products, meat products and seafood products were tested using the Chi2 test. Significance levels: ****: p < 1.10- 5 ; ***: p < 1.10-4; **: p < 1.10-3; *: p < 0.05. Only positive associations are shown. NA: Not appropriate.

**File Name: Supplementary Data 2**

**Description:** Strains used for assessment of growth, biofilm formation and colonization capacities The set of 42 strains representative of CC1, CC2, CC4, CC6, CC9, and CC121 (7 strains per CC) used for the experiments performed in this study are listed. CCs, cgMLST types and origins of the strains are indicated9,10. Phenotype of each strain regarding growth and biofilm formation in presence of 1.5 to 10 mg/L of benzalkonium chloride (BC) is indicated, as well as the presence (“Present”) and absence (“0”) of BC tolerance genes (qac, bcrABC, emrC and mdrL/ladR). mdrL and ladR are present in all isolates, as they are part of the Lm core genome. qacC, emrE and qacA are absent in all listed isolates. Tolerance/sensitivity of each strain, as deduced based on the presence/absence of BC tolerance genes is shown. In the “BC phenotype (observed)” column, dashes mean no difference of phenotype as compared to the absence of BC. BC: Benzalkonium chloride.

**File Name: Supplementary Data 3**

**Description:** Genes significantly associated with a dairy origin A genome wide association study was performed in order to identify genes significantly associated with dairy or meat origins among a set of 21,546 gene families of a pangenome built from 1,129 genomes. The table shows the 455 gene families significantly associated with a dairy origin, i.e. which obtained a Bonferroni-corrected p-value < 0.05 and an odd ratio > 1 for dairy products. All parameters provided in the scoary78 output are indicated.

**File Name: Supplementary Data 4**

**Description:** Genes significantly associated with a meat origin A genome wide association study was performed in order to identify genes significantly associated with dairy or meat origins among a set of 21,546 gene families of a pangenome built from 1,129 genomes. The table shows the 916 gene families significantly associated with a meat origin, i.e. which obtained a Bonferroni-corrected p-value < 0.05 and an odd ratio > 1 for meat products. All parameters provided in the scoary78 output are indicated.
