# Supplementary material for: Hypervirulent Listeria monocytogenes clones’ adaption to mammalian gut accounts for their association with dairy products
Source: Nat Commun. 2019 Jun 6;10:2488. doi: 10.1038/s41467-019-10380-0 (PMC6554400; doi:10.1038/s41467-019-10380-0)
Supplement: Supplementary file 7 — Reporting Summary [file 41467_2019_10380_MOESM7_ESM.pdf]

nature research

Corresponding author(s): Prof. Marc Lecuit

Last updated by author(s): Apr 11, 2019

Reporting Summary

Nature Research wishes to improve the reproducibility of the work that we publish. This form provides structure for consistency and transparency in reporting. For further information on Nature Research policies, see Authors & Referees and the Editorial Policy Checklist.

Statistics

For all statistical analyses, confirm that the following items are present in the figure legend, table legend, main text, or Methods section.

n/a	Confirmed	
☐	☒	The exact sample size (*n*) for each experimental group/condition, given as a discrete number and unit of measurement
☐	☒	A statement on whether measurements were taken from distinct samples or whether the same sample was measured repeatedly
☐	☒	The statistical test(s) used AND whether they are one- or two-sided<br>*Only common tests should be described solely by name; describe more complex techniques in the Methods section.*
☐	☒	A description of all covariates tested
☐	☒	A description of any assumptions or corrections, such as tests of normality and adjustment for multiple comparisons
☐	☒	A full description of the statistical parameters including central tendency (e.g. means) or other basic estimates (e.g. regression coefficient) AND variation (e.g. standard deviation) or associated estimates of uncertainty (e.g. confidence intervals)
☐	☒	For null hypothesis testing, the test statistic (e.g. *F*, *t*, *r*) with confidence intervals, effect sizes, degrees of freedom and *P* value noted<br>*Give P values as exact values whenever suitable.*
☒	☐	For Bayesian analysis, information on the choice of priors and Markov chain Monte Carlo settings
☒	☐	For hierarchical and complex designs, identification of the appropriate level for tests and full reporting of outcomes
☒	☐	Estimates of effect sizes (e.g. Cohen's *d*, Pearson's *r*), indicating how they were calculated

Our web collection on statistics for biologists contains articles on many of the points above.

Software and code

Policy information about availability of computer code

Data collection	No software was used for data collection
Data analysis	Bionumerics version 7.6.3 was used for single linkage clustering based on cgMLST profiles of isolates and for deduction of MLST clones based on PFGE profiles of isolates.<br>CLC Assembly Cell version 4.3.0 and SPADES version 3.11.0 were used to assemble genomic sequences.<br>BLAST+ v. 2.6.0 was used to detect BC tolerance genes and stress resistance genes.<br>Prokka version 1.12 was used to annotate genome sequences.<br>Roary version 3.6 was used for pangenome definition.<br>Scoary version 1.6.10 was used to look for genes associated with dairy or meat origins.<br>R was used for statistical analyzes (Mann-Whitney U test, AUC calculations, non-parametric tests) and graphical representations.

For manuscripts utilizing custom algorithms or software that are central to the research but not yet described in published literature, software must be made available to editors/reviewers. We strongly encourage code deposition in a community repository (e.g. GitHub). See the Nature Research guidelines for submitting code & software for further information.

Data

Policy information about availability of data

All manuscripts must include a data availability statement. This statement should provide the following information, where applicable:

- Accession codes, unique identifiers, or web links for publicly available datasets
- A list of figures that have associated raw data
- A description of any restrictions on data availability

The authors declare that all the data supporting the findings of this study are available within the article and its supplementary information files. Genome data analyzed in this study were generated in the context of the epidemiological surveillance of listeriosis in France and should not be made publicly available for regulatory issues, but can be available from the corresponding author upon reasonable request.

Field-specific reporting

Please select the one below that is the best fit for your research. If you are not sure, read the appropriate sections before making your selection.

☐ Life sciences ☐ Behavioural & social sciences ☒ Ecological, evolutionary & environmental sciences

For a reference copy of the document with all sections, see nature.com/documents/nr-reporting-summary-flat.pdf

Ecological, evolutionary & environmental sciences study design

All studies must disclose on these points even when the disclosure is negative.

Study description	We studied the totality of the food (n = 3,333) and clinical (n = 3,308) non-redundant isolates prospectively collected for 12 consecutive years (from 2005 and 2016) in the context of the surveillance of listeriosis in France. With this, we analysed the distribution of Lm sub-populations (clones) in different food products.<br>We then tested some phenotypes in-vitro and in-vivo in order to understand the differences of distribution of clones in the different types of food products. Seven genetically diverse strains per clone were tested in each experiement; each analysis was repeated at least three times for each isolate.<br>We also performed genomic analyzes by using all available genomes in our database (n = 2,928) in order to detect an unrichement of disinfectant and stress resistance genes in some clones.
Research sample	We analyze a collection of Listeria monocytogenes isolates collected in the context of the epidemiological surveillance of listeriosis in France.<br>This collection has a high level of exhaustiveness due to mandatory declaration of listeriosis in France and is therefore highly representative of the listeriosis cases that occurred in France during the study period. Regarding the food isolates, 2.623 were collected in the context of food alerts, which are triggered when contaminated food products are on the market. The remaining 710 isolates (21.3%) were collected in the context of own-checks performed by food industries or in case of investigations following neurolisteriosis cases. Therefore, they largely represent the Lm isolates circulating in France, to which the population is exposed.
Sampling strategy	For the 3,308 clinical isolates included, they correspond to all clinical isolates collected at the CNRL between January 2005 and May 2016. This collection has a high level of exhaustiveness due to mandatory declaration of listeriosis in France and is therefore highly representative of the listeriosis cases that occurred in France during the study period. Regarding the food isolates, 2.623 were collected in the context of food alerts, which are triggered when contaminated food products are on the market. The remaining 710 isolates (21.3%) were collected in the context of own-checks performed by food industries or in case of investigations following neurolisteriosis cases. Therefore, they largely represent the Lm isolates circulating in France, to which the population is exposed.
Data collection	Isolates were collected at the National Reference Centre for Listeria (NRCL) in the context of the French surveillance of listeriosis between January 2005 and May 2016.
Timing and spatial scale	Isolates were collected between January 2005 and May 2016 with no interruption.
Data exclusions	This collection of isolates was deduplicated in order to avoid any bias in the analyses. More specifically, only one isolate was considered in case of maternal-neonatal listeriosis (mother's isolates were kept). Regarding the food isolates, only one was considered when several had identical cgMLST type or CC (cf. methods below) and identical food alert number or precise food product.
Reproducibility	Regarding experimental procedures, each clone of interest was represented by 7 genetically diverse isolates, which were tested independently at least three times.
Randomization	No randomization was applied, as we work on an exhaustive collection of isolates.
Blinding	No blinding was applied, as we work on an exhaustive collection of isolates.

Did the study involve field work? ☐ Yes ☒ No

Reporting for specific materials, systems and methods

We require information from authors about some types of materials, experimental systems and methods used in many studies. Here, indicate whether each material, system or method listed is relevant to your study. If you are not sure if a list item applies to your research, read the appropriate section before selecting a response.

Materials & experimental systems

n/a	Involved in the study
☒	☐ Antibodies
☒	☐ Eukaryotic cell lines
☒	☐ Palaeontology
☐	☒ Animals and other organisms
☒	☐ Human research participants
☒	☐ Clinical data

Methods

n/a	Involved in the study
☒	☐ ChIP-seq
☒	☐ Flow cytometry
☒	☐ MRI-based neuroimaging

Animals and other organisms

Policy information about studies involving animals; ARRIVE guidelines recommended for reporting animal research

Laboratory animals	We used 7- to 8-week-old mEcad E16P KI female mice in a C57BL/6J genetic background (Disson et al., Modeling human listeriosis in natural and genetically engineered animals. Nat Protoc 4, 799-810 (2009)
Wild animals	No wild animals were used
Field-collected samples	The study did not involve field-collected animals
Ethics oversight	All the procedures used in this study are in agreement with the guidelines of the European Commission for the handling of laboratory animals, directive 86/609/EEC. They were approved by the ethical committee of Institut Pasteur (CETEA-C2EA no. 89) under the number dap170057 and received an agreement from the ministry of higher education, research and innovation under the number APAFIS#14644-2018041116183944 v1.

Note that full information on the approval of the study protocol must also be provided in the manuscript.
